# Supplementary figures and images for: Unveiling the Mycodrosophila projectans (Diptera, Drosophilidae) species complex: Insights into the evolution of three Neotropical cryptic and syntopic species
Source: PLoS One. 2022 May 25;17(5):e0268657. doi: 10.1371/journal.pone.0268657 (PMC9132268; doi:10.1371/journal.pone.0268657)

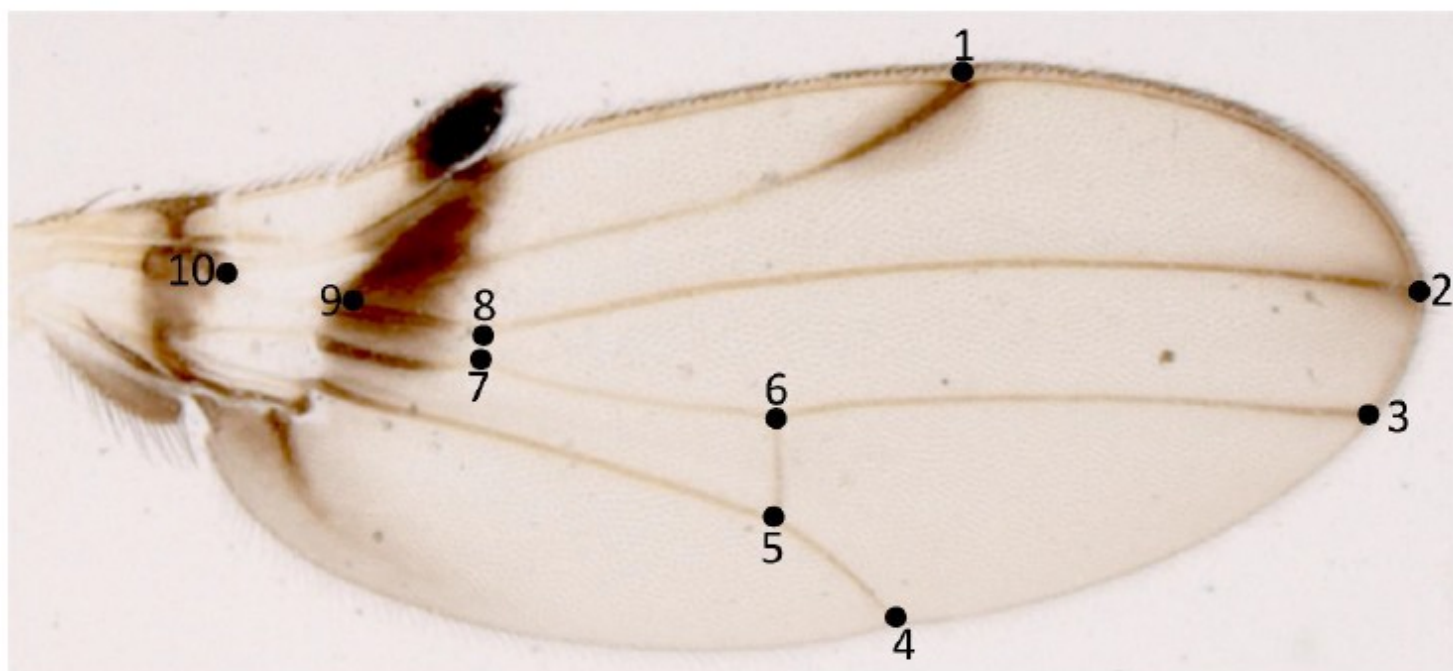

Supplement: S1 Fig — (PDF) [file pone.0268657.s001.pdf]

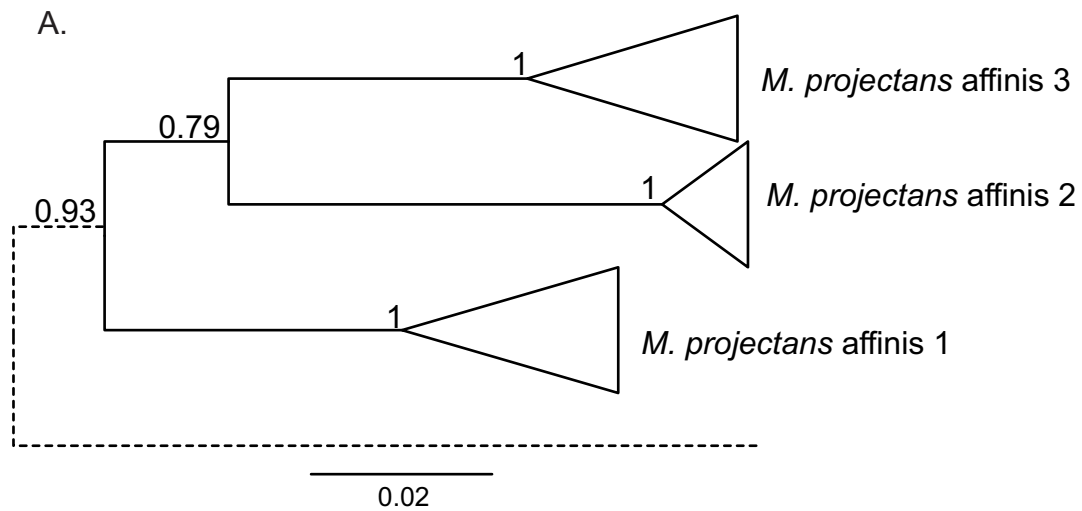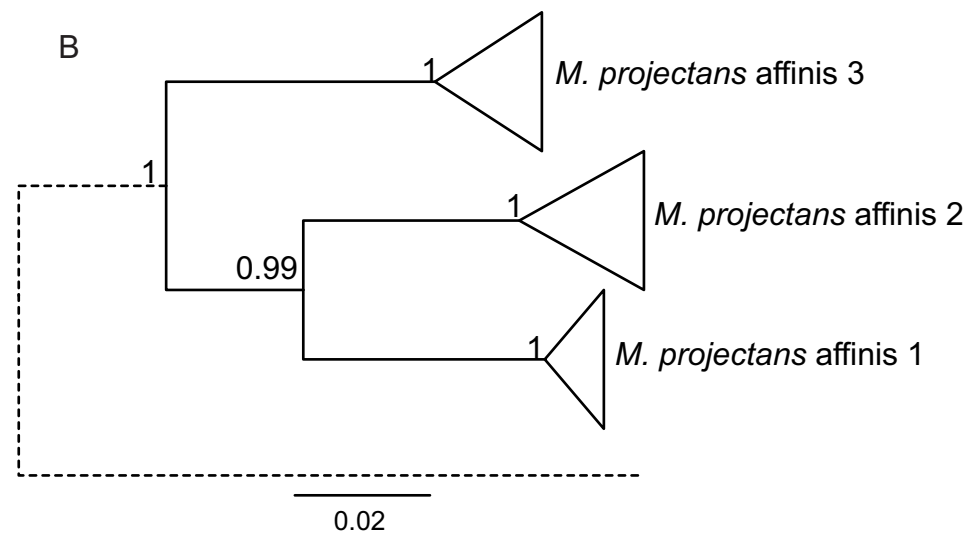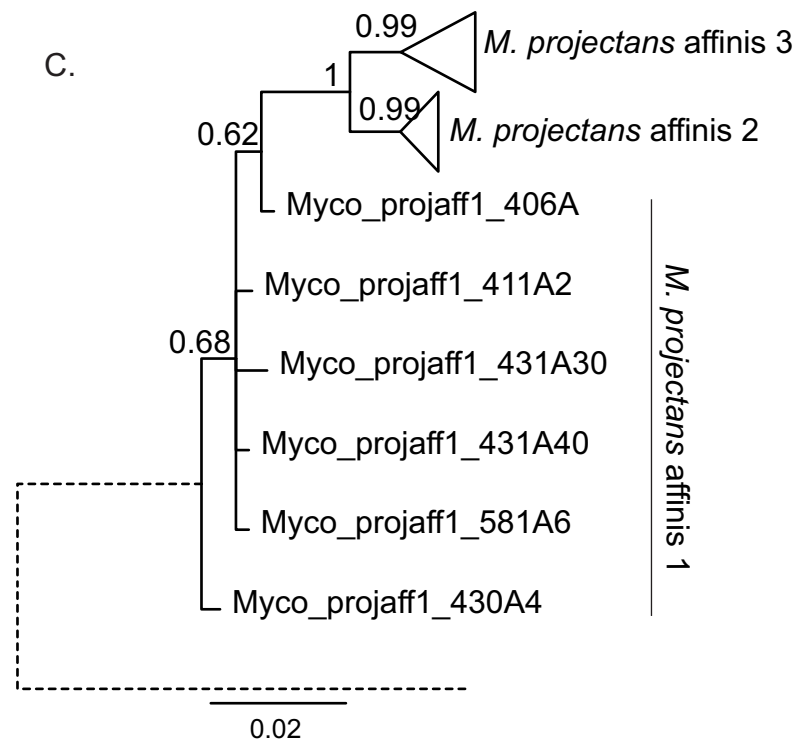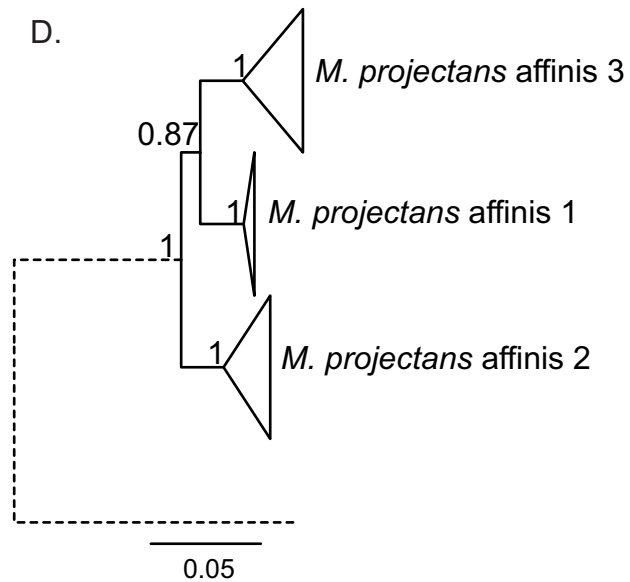

Supplement: S2 Fig — Majority rule consensus tree recovered through Bayesian analysis based on (A) COI, (B) COII, (C) HB, and (D) AMD datasets. Branch lengths are proportional to the scale, given in substitutions per site. Values near the internal branches represent the posterior probability (PP) of each clade. (PDF) [file pone.0268657.s002.pdf]

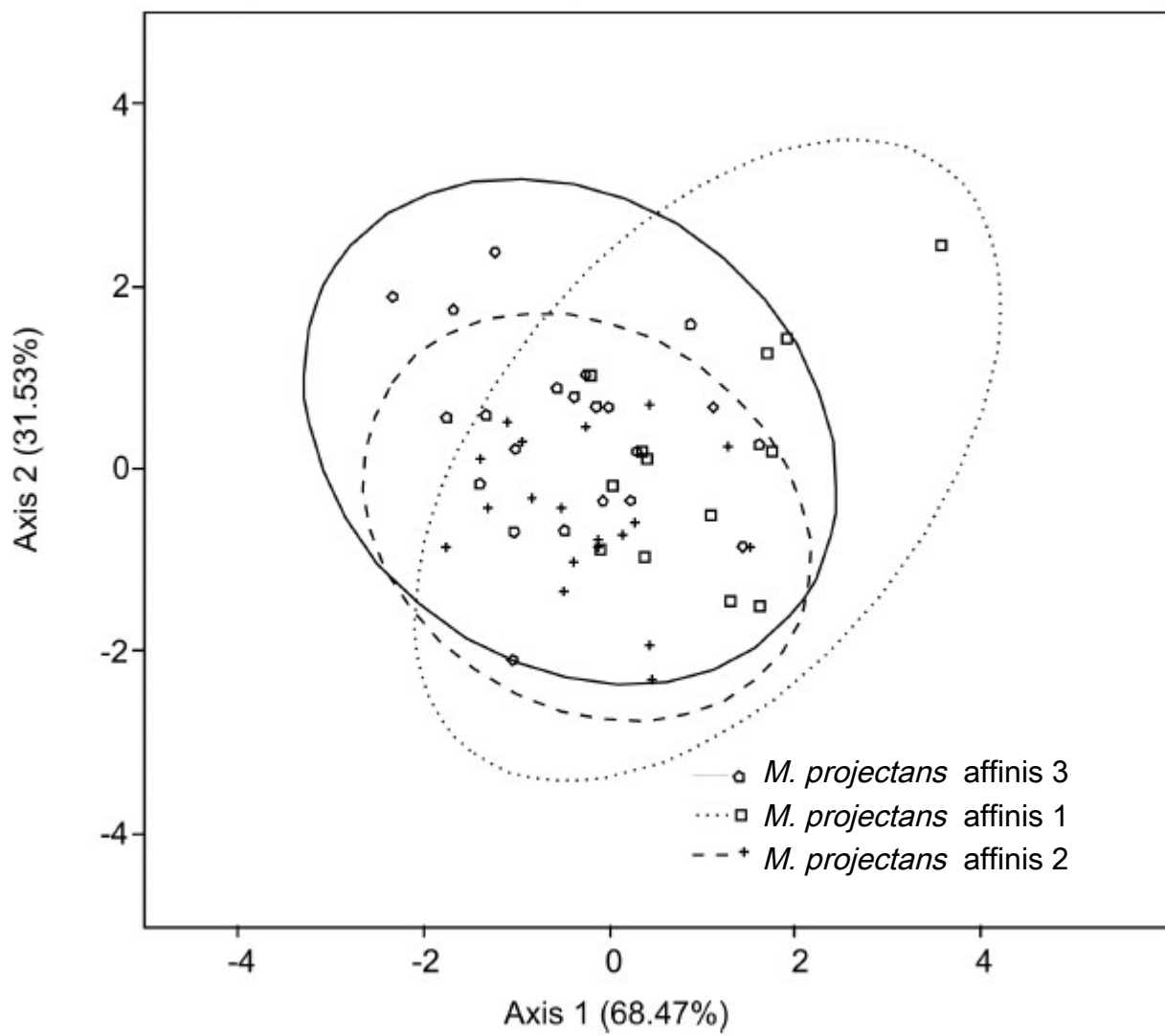

Supplement: S3 Fig — (PDF) [file pone.0268657.s003.pdf]

*M. projectans* affinis 1

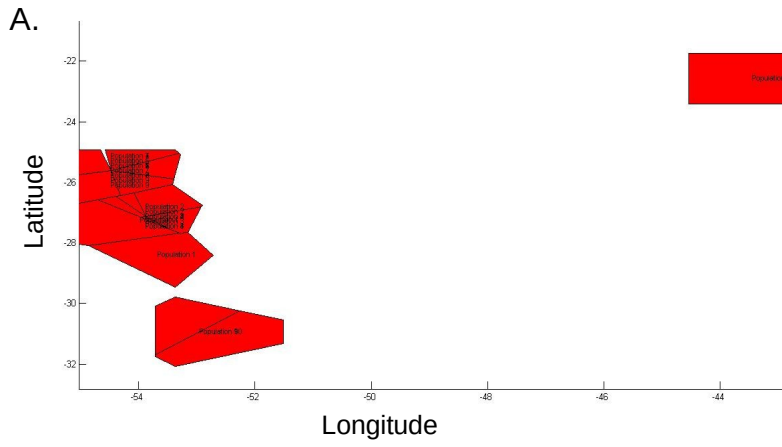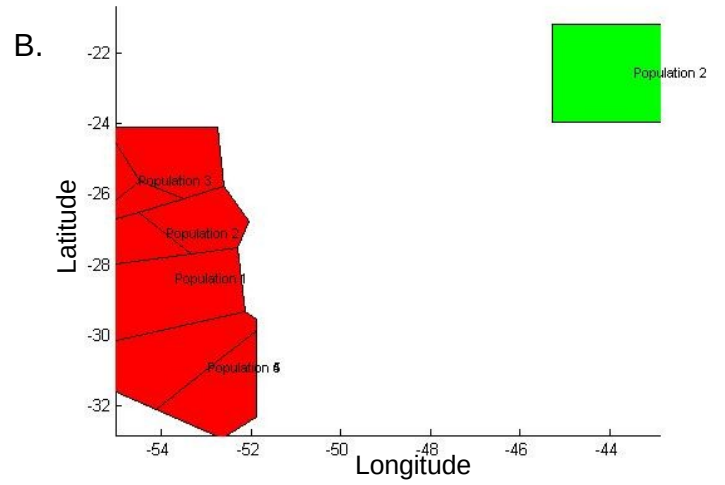

*M. projectans* affinis 2

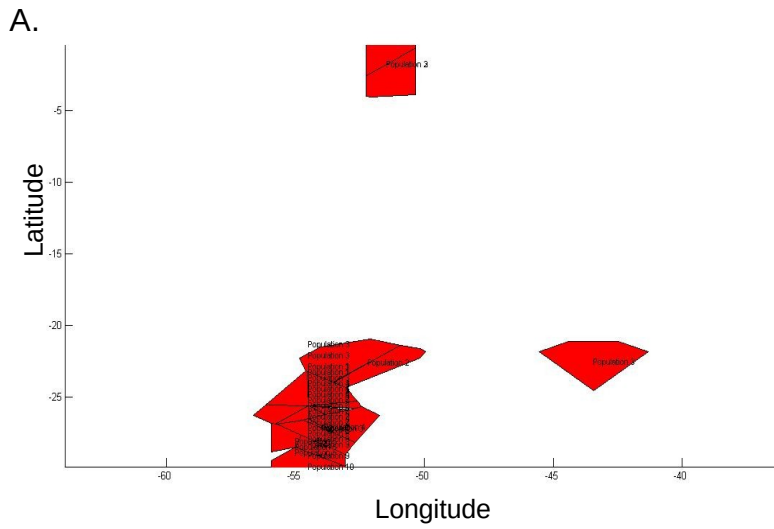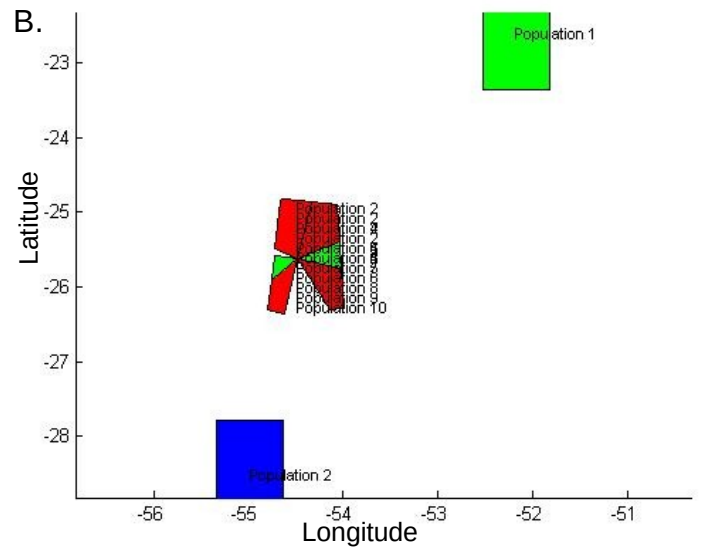

*M. projectans* affinis 3

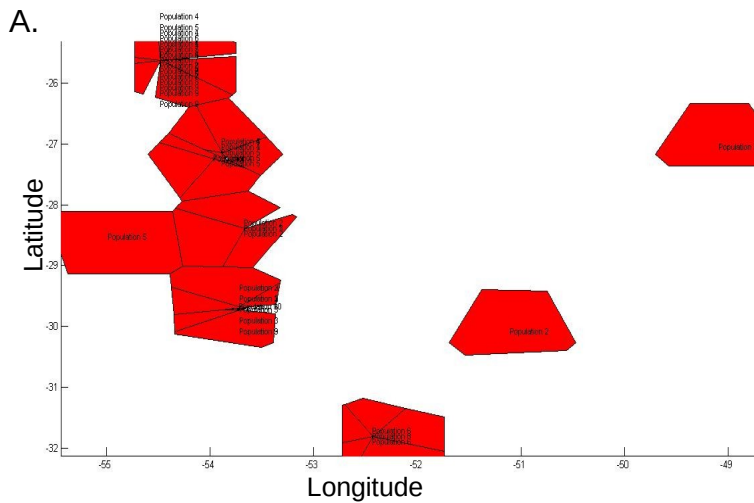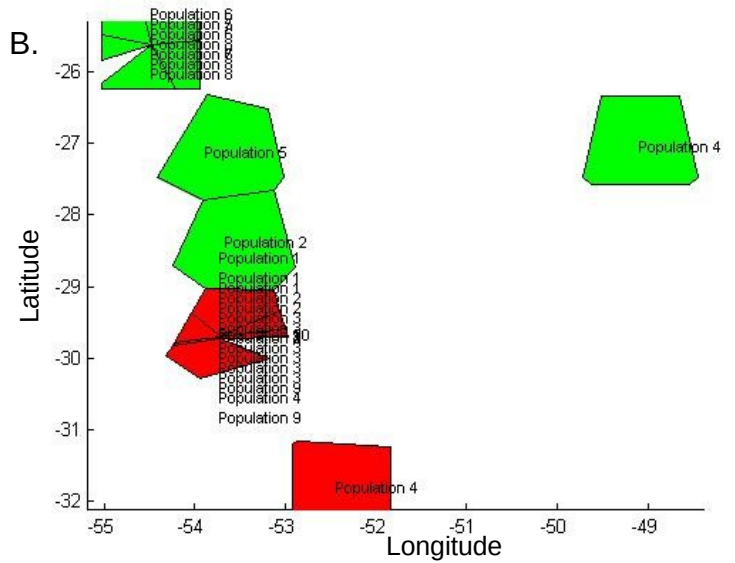

Supplement: S6 Fig — Results from the spatial clustering analysis performed by BAPS for each of the three species of the M. projectans complex based on (A) COI, and (B) COII datasets. Axis x and y reflect the longitude and latitude of each population, whereas each color represents a different cluster of populations. (PDF) [file pone.0268657.s006.pdf]

A. *M. projectans* affinis 2

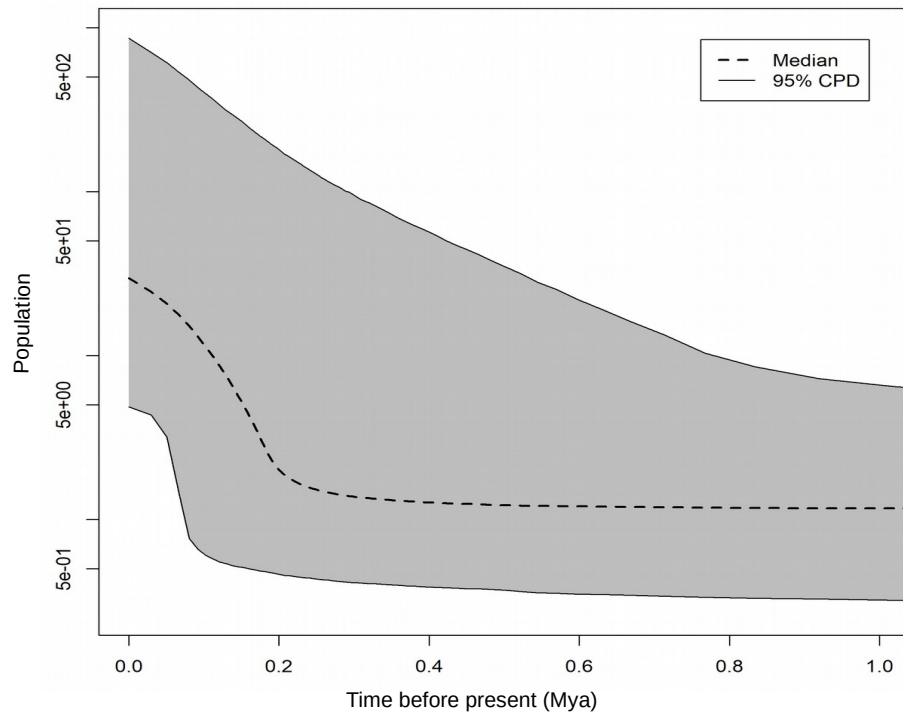

B. *M. projectans* affinis 3

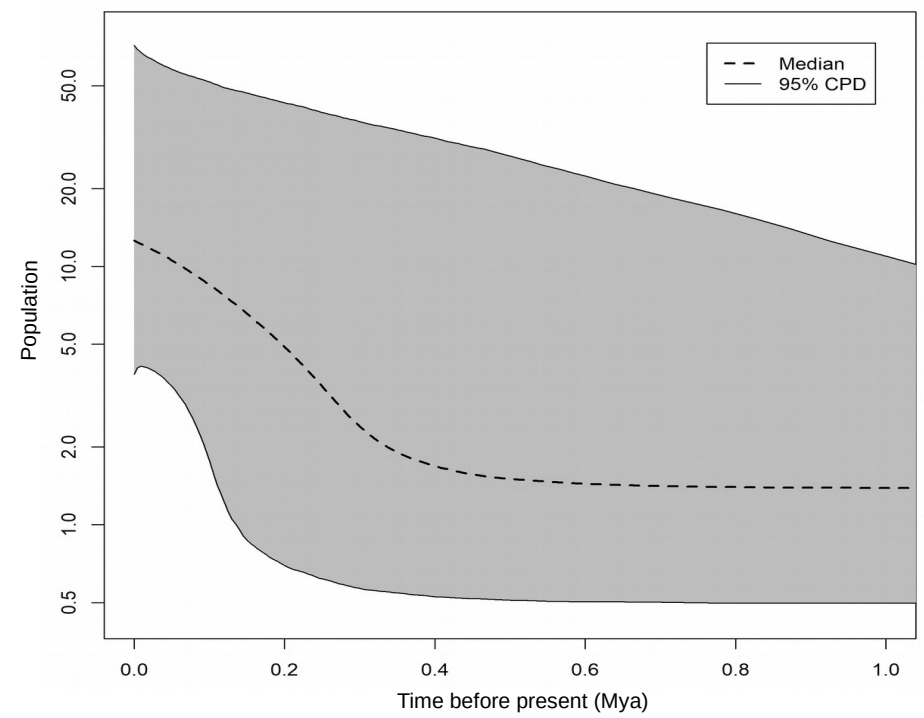

Supplement: S7 Fig — Extended Bayesian Skyline Plots depicting oscillations of population sizes faced by M. projectans affinis 2 (A) and M. projectans affinis 3 (B) during the last 1 Mya. (PDF) [file pone.0268657.s007.pdf]
