# Supplementary material for: Unveiling the Mycodrosophila projectans (Diptera, Drosophilidae) species complex: Insights into the evolution of three Neotropical cryptic and syntopic species
Source: PLoS One. 2022 May 25;17(5):e0268657. doi: 10.1371/journal.pone.0268657 (PMC9132268; doi:10.1371/journal.pone.0268657)
Supplement: S4 Fig — (A) M. projectans affinis 1, (B) M. projectans affinis 2, and (C) M. projectans affinis 3. Each circle represents a different haplotype, whose size is proportional to frequency. Each color represents different sampling points, in accordance with the legend presented on Fig 1. Black small circles represent median vectors. Dashes in the lines connecting different haplotypes represent the number of mutations between them. (PDF) [file pone.0268657.s004.pdf]

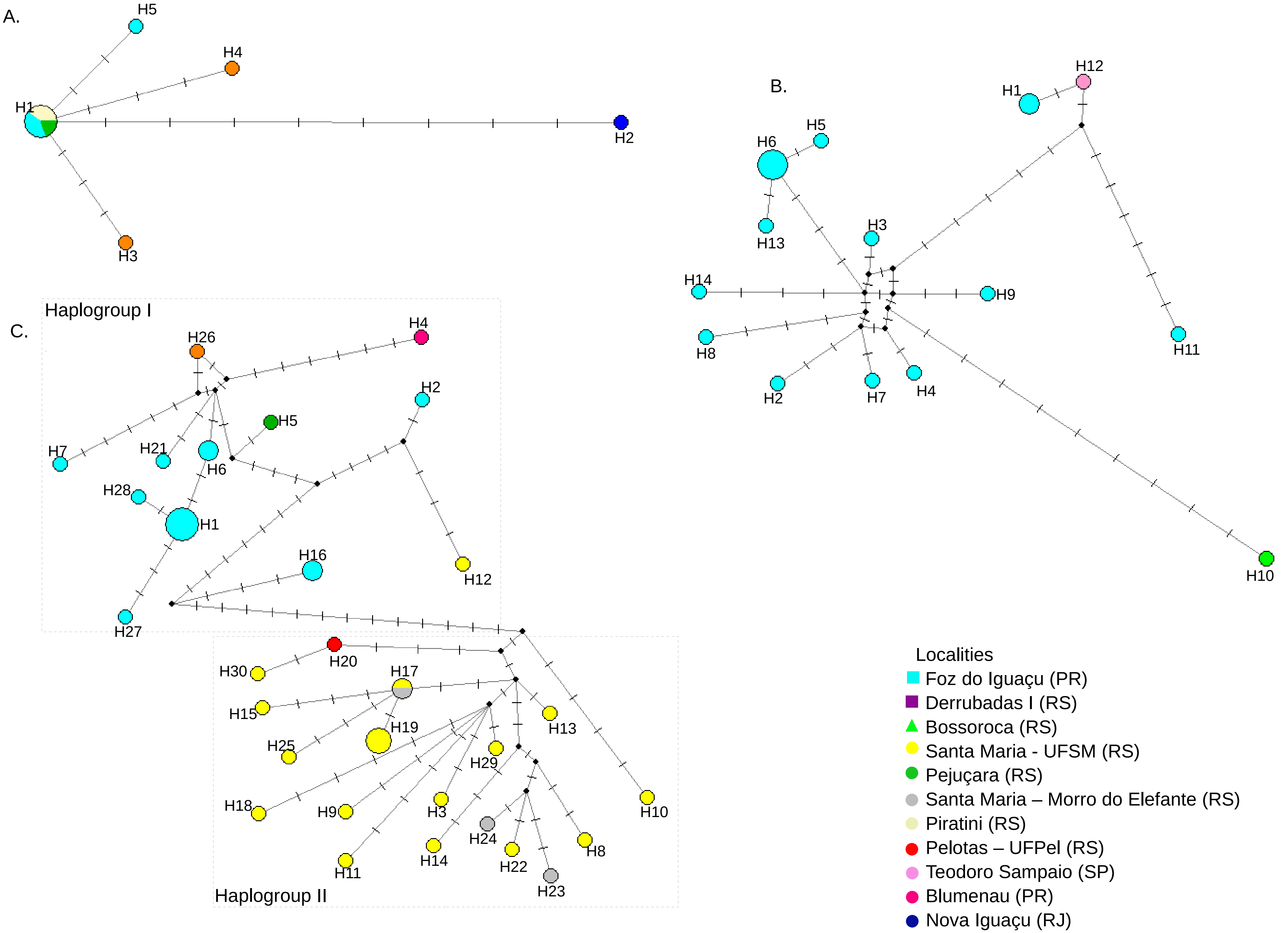
A.
H5
H4
H1
H2
H3
B.
H12
H1
H5
H6
H13
H3
H14
H9
H8
H2
H7
H4
H11
H10
C.
Haplogroup I
H4
H26
H2
H5
H7
H21
H6
H28
H1
H16
H12
H27
H30
H20
H17
H15
H19
H25
H13
H29
H18
H9
H3
H10
H24
H14
H8
H22
H11
H23
Haplogroup II
Localities
Foz do Iguaçu (PR)
Derrubadas I (RS)
Bossoroca (RS)
Santa Maria - UFSM (RS)
Pejuçara (RS)
Santa Maria – Morro do Elefante (RS)
Piratini (RS)
Pelotas – UFPel (RS)
Teodoro Sampaio (SP)
Blumenau (PR)
Nova Iguaçu (RJ)
