# Supplementary material for: Unveiling the Mycodrosophila projectans (Diptera, Drosophilidae) species complex: Insights into the evolution of three Neotropical cryptic and syntopic species
Source: PLoS One. 2022 May 25;17(5):e0268657. doi: 10.1371/journal.pone.0268657 (PMC9132268; doi:10.1371/journal.pone.0268657)
Supplement: S5 Fig — Median-joining network reconstructed with (A) HB, and (B) AMD phased alleles characterized for each of the three species of the M. projectans complex. Each circle represents a different haplotype, whose size is proportional to frequency. Each color represents different sampling points, in accordance with the legend presented on Fig 1. Black small circles represent median vectors. Dashes in the lines connecting different haplotypes represent the number of mutations between them. (PDF) [file pone.0268657.s005.pdf]

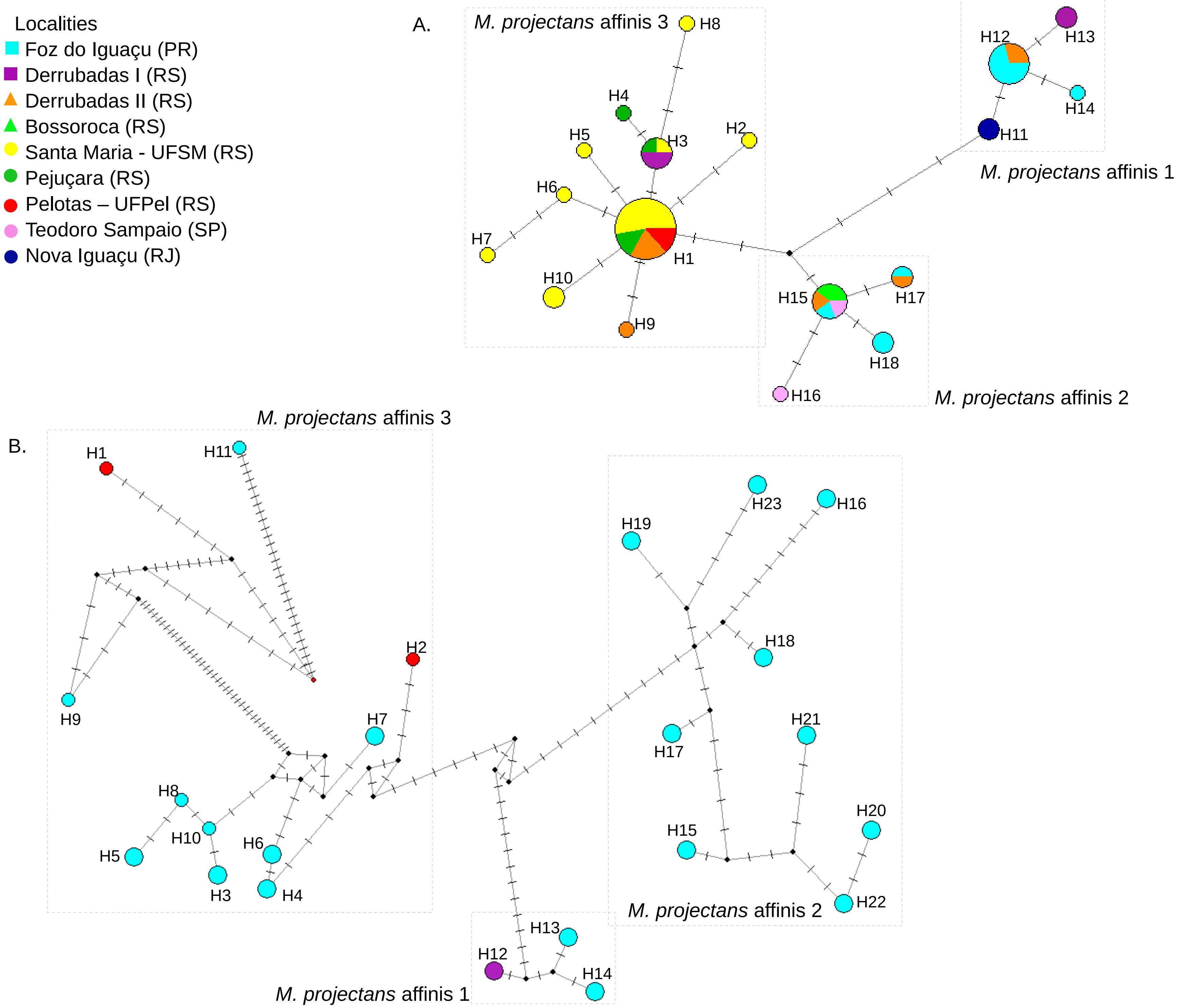

Localities
Foz do Iguaçu (PR)
Derrubadas I (RS)
Derrubadas II (RS)
Bossoroca (RS)
Santa Maria - UFSM (RS)
Pejuçara (RS)
Pelotas – UFPel (RS)
Teodoro Sampaio (SP)
Nova Iguaçu (RJ)
A.
M. projectans affinis 3
H8
H4
H5
H3
H2
H6
H7
H1
H10
H9
H12
H13
H14
H11
M. projectans affinis 1
H15
H17
H18
H16
M. projectans affinis 2
B.
M. projectans affinis 3
H1
H11
H2
H9
H7
H8
H10
H5
H3
H6
H4
H19
H23
H16
H18
H17
H21
H15
H20
H22
M. projectans affinis 2
H13
H12
H14
M. projectans affinis 1
