# Supplementary material for: Unveiling the Mycodrosophila projectans (Diptera, Drosophilidae) species complex: Insights into the evolution of three Neotropical cryptic and syntopic species
Source: PLoS One. 2022 May 25;17(5):e0268657. doi: 10.1371/journal.pone.0268657 (PMC9132268; doi:10.1371/journal.pone.0268657)
Supplement: Attachment — Submitted filename: Response to Reviewers.pdf [file pone.0268657.s025.pdf]

Santa Maria, March 08th, 2022.

Dear Dr. William J. Etges,

Please find attached the MS entitled **"Unveiling the Mycodrosophila projectans (Diptera, Drosophilidae) species complex: insights into the evolution of three Neotropical cryptic and syntopic species"** which contains a revised version of the previous submission to PLOS ONE (PONE-D-21-28403). We thank you for the opportunity as well as for the constructive criticism, which allowed several improvements in our study.

We carefully followed the recommendations and provide below the explanation (in bold) on how we proceeded about each specific comment (in italics). Excerpts from the text are represented in yellow in this letter, and are highlighted in the tracked version of the MS.

We are confident the current version is much improved and hope it reaches the level of quality expected by PLOS ONE.

Sincerely,

L. J. Robe, on behalf of all authors

Editor`s comment:

"The authors have done a good job in responding to the peer reviewers' comments, but the authors did not follow the instructions listed in the initial decision letter to address all of the English language problems. I think the paper contains some interesting and verifiable results, but the Discussion needs to be shortened and all speculation and redescriptions of the Results section need to be eliminated. I have uploaded an edited copy of your MS with many specific changes and questions. Several sections and paragraphs in the Discussion section should be deleted. Overall, the writing style is wordy with much jargon. It reads like a thesis. Again, I suggest the MS be proofread by an English speaker before resubmission because I may not have found all of the grammar and wording problems."

Thank you for your effort in thoroughly reviewing our manuscript. All the changes pointed out in the pdf were performed, and the MS was proofread by a specialized company (see certificate attached to the submission). We also withdrawn several speculations of the Discussion, as well as the redescriptions of the results. Thus, the last section was significantly shortened.

Concerning each commentary presented in the pdf:

Abstract – line 39: *Look up the definition of niche.*

As suggested, we withdrawn the reference to "biotic and abiotic niches", so that the mentioned sequence now looks like: "Ecologically, sympatry and syntopy seem to be recurrent for these three cryptic species, which present widely overlapping niches, implying niche conservatism." (lines 34-36)

Introduction – lines 69-71:

"This last sentence needs rewriting or deletion. There are many reasons beyond non-visual signals why cryptic species exist. Why is vision so important? All kinds of signaling systems are known. They could have diverged in allopatry and then converged later."

We decided to rewrite the sentence, as follows: "The mechanisms responsible for this pattern are not well understood [13], but it has been suggested that sexual selection acting at the level of non-visual signals [14], recent divergence [15], chance fixation of alleles related to epistatic incompatibilities [16], and allopatric speciation associated with morphological stasis or convergent evolution [17] may help to explain the ubiquity of this phenomenon." (lines 56-60)

Materials and Methods – line 139: *State what you mean by "active period".*

The sentence was rewritten to: "In these localities, active searches for fruiting bodies of macroscopic fungi were performed in forest fragments during daylight." (lines 116-118)

Results – line 357: *What is "Bold system"? Define*.

BOLD is defined in line 164 of the Materials and Methods: "All individuals were identified by the DNA barcoding in BOLD (Barcode of Life Data) System [51] using COI sequences."

Results – title presented in lines 433-433: replace "What can we say about the differential patterns of genetic diversity and abiotic niches that distinguish the three species and what this suggests about their speciation?" to "Patterns of genetic diversity and abiotic niches distinguish the three species and suggest modes of speciation".

The title was rewritten to: "Patterns of distribution of genetic diversity and abiotic niches help to disentangle evolutionary history and suggest modes of speciation" (lines 375-377)

Discussion – lines 569-591: *These are a restatemnt of the results. Delete.*

The paragraph was withdrawn, and only two sentences were left to mention the cryptic morphology: "The cryptic morphology among the three species was confirmed after rigorous analysis, and only a few subtle morphological differences were found for the three species in terms of aedeagus morphology and abdominal color patterns. However, none of these allowed the three species of the complex to be unambiguously distinguished" (lines 499-502). Otherwise, this aspect would not be mentioned in the discussion, and there would be a gap to the comprehension of the next paragraphs.

Discussion – lines 595-596, 600-601: *Previous sentence deleted. Its irrelevant and speculative.*

Both sentences were deleted, as well as all the section dealing with preferential mating. The new section now looks like: "In addition to this cryptic morphology, the M. projectans complex also detaches for recurrent sympatry and syntopy among its species, as observed here in three samples from the Atlantic Forest and the Pampa biome. The coexistence of cryptic species seems to be a common pattern in Mycodrosophila since Lacy [92] also reported syntopy for two cryptic species in the Nearctic (M. claytonae A and B). Within the Zygothrica genus group, the co-occurrence of cryptic species has also been reported for Neotropical species of the genera Zygothrica and Hirtodrosophila [28, 93]" (lines 502-508). This paragraph was further associated with the next one, continuing with: "Nevertheless, for the M. projectans complex, beyond co-occurrence, patterns of biotic and abiotic niches were also quite similar, and species used the same genera of fungi as breeding sites. Despite differences among species concerning the amplitude of potential distribution, neither pairwise comparison between abiotic niches recovered through ENM strategies rejected the null hypothesis of niche equivalency. These results support the hypothesis of niche conservatism for the M. projectans complex [29], suggesting that it may be a common pattern in the Zygothrica genus group. Nevertheless, further ecological studies are necessary to clarify in which way these species use their resources and how competition affects their co-occurrence" (lines 509-517).

Lines 624-61, and 632-637 were also deleted, and the two next paragraphs were shortened and associated: "Patterns of morphological similarity and niche equivalency seem to have been maintained despite ancient divergence. In this sense, although there were incongruences among gene trees regarding the topology of the relationships within the M. projectans complex, the multi-locus strategy used to reconstruct the

species tree under a relaxed molecular clock strategy allowed us to date the two cladogenesis events to approximately 17–15 Mya ago. These estimates suggest that the diversification of the M. projectans complex occurred in the Neogene period during the Miocene [94]. This contrasts with the pattern commonly found for Drosophilidae, in which cryptic species encompass recently diverged lineages [93, 95, 96] that did not have enough time to accumulate diagnosable features. Thus, this study provides an interesting example of morphological conservation, despite ancient divergence. Furthermore, this ancient divergence, in view of the similarity of niches evidenced here for the three lineages, allows us to hypothesize that divergence of the species occurred in allopatry or parapatry, with current records of sympatry and syntopy reflecting secondary contact. This hypothesis of allo- or parapatric distribution in more ancient times was also supported by EBSP analyses, which showed contrasting patterns of demographic fluctuations for all species. In fact, whereas M. projectans affinis 1 did not present significant signals of population expansion, such signals were supported for M. projectans affinis 2 and M. projectans affinis 3 as occurring at different moments of the Middle Pleistocene [94], ca. 200 and 400 Kya, respectively. Such differences would certainly not be expected if species were sympatric, especially in the face of straightforward evidence of niche equivalence" (lines 518-537)

Discussion, line 655-656: Replace "these three species seem to have expanded to the sampled region quite recently" to "these three species seem to have expanded quite recently". **In this sentence, we preferred to adopt an intermediate version stating that "these three species seem to have expanded to Southern Brazil quite recently" (lines 538-539), since our modelling results clearly suggest an expansion to this specific area.**
