# Supplementary material for: Unveiling the Mycodrosophila projectans (Diptera, Drosophilidae) species complex: Insights into the evolution of three Neotropical cryptic and syntopic species
Source: PLoS One. 2022 May 25;17(5):e0268657. doi: 10.1371/journal.pone.0268657 (PMC9132268; doi:10.1371/journal.pone.0268657)
Supplement: Attachment — Submitted filename: _Response to the reviewers.docx [file pone.0268657.s023.docx]

Dear Dr. William J. Etges,

Please find attached the MS entitled **“Unveiling the Mycodrosophila projectans (Diptera, Drosophilidae) species complex: insights into the evolution of three Neotropical cryptic and syntopic species”** which contains a revised version of the previous submission to PLOS ONE (PONE-D-21-28403). We thank you for the opportunity as well as for the constructive criticism from reviewers, which allowed several improvements in our study.

We followed carefully the recommendations and provide below a point-by-point explanation (in bold) on how we proceeded about each specific comment of the reviewers (in italics). As recommended, we also provide a revised manuscript and an additional manuscript file where all the changes made are highlighted.

Concerning deposition of data in repositories, all new sequences were submitted to GenBank, and accession numbers are provided in Table S2. These sequences will be directly released once they appear on a published document, according to NCBI standards.

The Neotropical shapefile employed on Figures 1 and 5 was created by Löwenberg-Neto (2014), which is accordingly cited on the figure’s legends. According to Löwenberg-Neto (2014), this “shapefile is freely available and may be downloaded at <http://purl.org/biochartis/neo2014shp>”. The Brazilian Biomes shapefiles employed on Figure 1 are the result of a cooperation signed between the *Instituto Brasileiro de Geografia e Estatística* (IBGE) and the Ministry of the Environment, two Brazilian government entities. It is also freely available and may be downloaded at https://www.ibge.gov.br/geociencias/cartas-e-mapas/informacoes-ambientais/15842-biomas.html?=&t=downloads.

We are confident the current version is much improved and hope it reaches the level of quality expected by PLOS ONE.

Sincerely,

L. J. Robe, on behalf of all authors

**Reviewer 1:**

**Reviewers Comments to the Author**

*The authors show that the species Mycodrosophila projectans consists of (at least) three cryptic species. They do so by using DNA barcode sequencing and excluding interferences from numts and hitchhiking mitochondrial DNA in endosymbiotic parasites. The authors found a surprizing result that, despite the three species being so similar and overlapping in their niches, they have diverged in two events, dating back 15 and 17 MYA. The authors offer an explanation that the divergence was soon followed by geographic isolation, and that these species later came back in contact through migration events, possibly driven by the scarcity of their mushroom hosts. The authors also found subtle morphological characters that can be used to distinguish the three species from each other, which can be useful for future field research with these species.*

***Critique:***
*1) This is a thorough study, using many analyses and techniques to understand that there are three species present, how they can be distinguished on the molecular levels and identifying morphological markers, leading to the identification of key features on the abdominal color pattern and the male genitalia. Although I have not performed these kinds of analyses myself, the results are, at least to me, convincingly demonstrated and explained. I like how the authors have tried to figure out the timing of divergence and the discrepancy between the species being comparatively old but still cryptic. It makes a nice story.
2) When I read through the paper, I was a bit lost until I got to the discussion, where finally everything was nicely explained. I think that the paper could gain some clarity in the results section if the results were not just written out, but if each paragraph could have the following logic, beginning with stating the question, then stating what you did, then what happened (this is what the Results section currently contains), followed by a brief statement what the results mean and stating what the next question therefore is. This pattern of writing gives a reader not so familiar with the methodology more clarity, especially important for the broad audience of PLoS ONE.
3) There were some minor punctuation, phrasing and other language errors, especially in the first half of the paper, which the editors can correct later in the process. I just want to point out one: cactophilic is spelled with an i after the letter h.
4) Otherwise well done!*

**We thank the reviewer for the motivating comments as well as for the suggestions to improve our manuscript. We have stated the initial question involving each of the different approaches on the Results section. We also corrected some grammar errors and misspellings that passed on the first version of the manuscript. We apologize for them.**

**Reviewer 2:**

**Reviewers Comments to the Author**

*I think your manuscript convincingly shows your samples of M. projectans consist of three reciprocally monophyletic species. Below are my comments on the manuscript. In general, there three issues that need to be addressed.*

*→ First, the grammatical errors should be corrected. I think I have found most of them (see below) but the manuscript should be re-checked.*

**We appreciate your suggestions to improve our manuscript. The grammatical errors were checked and corrected. Thank you for your detailed review on this aspect.**

*→ Second, the discrepancy in sequencing samples across species (and loci) should be explained in the text. The discrepancy would likely not affect conclusions, but the method of choosing samples should be described. Were samples for sequencing randomly chosen from each geographic location? Was it based on tentative morphological species delimitation? Was it simply all males collected? Why the discrepancy between mtDNA and nucDNA sample sizes? Was there failure of amplification for some specimens?*

**These discrepancies are now addressed on the manuscript, where we added the following sentences:**

1) **Lines 136-138: “Just males were employed in further analyses because morphological differences in Drosophilidae are frequently described or even found only through inspection of the internal genital structures of males.”**
2) **Lines 327-328: “****Sampling disparity occurred due to frequent failures of amplification, that commonly led to the premature termination of some DNA samples.”**

*→ And three,* *Wolbachia is importantly discussed as a factor that could disrupt reciprocal monophyly of species, and I think the evidence presented convincingly shows this is not the case for these species. However, I* *think a simple PCR diagnostic for Wolbachia infection is warranted here to support this conclusion. Is there a known Wolbachia infection in these species? At the very least, there should be some discussion as to what is known about Wolbachia incidence and/or prevalence in these or closely related species. There are several additional minor comments included among the comments below.*

**Further information about Wolbachia and further interpretation of the results were added to the text:**

1) **In the Introduction, lines 103-106: “Furthermore, as the endoparasite Wolbachia has already been reported in some groups of mycophagous drosophilids, including one species of Hirtodrosophila, and as several species of insects can occur in a single fungus [29], mushrooms can be considered a hotspot for horizontal transfer of this endoparasite.”**
2) **In the Discussion, lines 537-554: “These results allowed rejecting the hypothesis that the clades observed by Machado et al. [25] would be a result of selective scanning associated with three different and recent invasions of Wolbachia in M. projectans [28]. Nevertheless, it is still possible that such patterns are resultant of ancient invasions independently fixed in different populations, that were first associated with cytoplasmic incompatibility, and post-zygotic isolation, and later enhanced the emergence or pre-zygotic isolation through reinforcement [91-93]. In this case, Wolbachia could act as a pro-speciation factor, and such a longstanding infection could result in fixation also for nuclear alleles. In fact, there is evidence for Wolbachia infection in at least one species of the Zygothrica genus group, and this endosymbiont was shown to be more frequent in mycophagous than non-mycophagous Diptera [29]. Although at a first sight simple diagnostic amplification could be used to confirm the presence of Wolbachia in our samples, shortages related to the amplification success presented by some of the markers employed here would not allow a confident description of prevalence patterns with such a methodology. Thus, it remains to be further evaluated if Wolbachia could or not be involved in the speciation of the M. projectans complex. Even so, this uncertainty does not affect the conclusion about the presence of at least three species under the general morphotype previously ascribed to M. projectans.”**

*P# refers to the paragraph number for the given section.*

***Intro:*** *P1: “ mechanisms responsible for these patterns ...” -> “mechanism responsible for this pattern” (i.e. cryptic variation)*

**Corrected.**

*P2: “using them as resource ...” -> “using them as a resource”*

**Corrected.**

*P4: Machado et. al is said to describe the same external morphotype for the projectans complex, then the next sentence describes the limitation of mtDNA markers. It should be explicitly stated that Machado et. al used a mtDNA marker to delimit species.*

**The sentence was changed to “However, when some of these Neotropical populations were evaluated through DNA Barcoding approaches using only nucleotide sequences of the mitochondrial cytochrome oxidase c subunit I (COI) gene, at least three different lineages were uncovered [25]: M. projectans, M. projectans affinis 1 and M. projectans affinis 2.” (lines 92-96).**

*P5: “These strategies allowed confirmation the existence ...” -> “confirmation of the existence ...”*
**This sentence was corrected to: “These strategies allowed us to confirm the existence”.**

*Methods:
Table 1: Was the reduced sampling of nuclear loci (and disparity between species) by choice or failure of amplification? A quick glance at table S2 makes it look like sequencing was guided by geographic location. This would likely not affect overall conclusions of the paper, but it should be addressed in the text.*

**The two questions are now addressed on the text:**

1) **The reduced sampling of nuclear markers is mostly related to amplification failures (see lines 327-328): “Sampling disparity occurred due to frequent failures of amplification, that commonly led to the premature termination of some DNA samples.”**
2) **Sequencing was not guided by geographic location, but by sampling success. Furthermore, only males were employed in morphological and molecular analyses, which further reduced sampling size (see lines 136-138):**

*Morphological and molecular approaches for species delimitation:
P3: How was convergence of the MCMC used with the Stacey analysis assessed?*

**This information was added on lines 214-215: “Convergence was evaluated in Tracer 1.60 [65], checking likelihood and posterior probability traces and confirming that all ESS values are above 200.”**

*Results:
Species delimitation by molecular approaches:
P1: “None coding sequences” -> “No coding sequences”*

**Corrected for “No sequence” since all sequences are coding.**

*P2: “revealed reciprocally monophyletic.” -> “revealed to be reciprocally monophyletic”*

**Corrected.**
 *Genetic Diversity, structure and demographic history:
P4: “Likewise, at the same ...found in the same point” -> “Likewise, in the same ...found at the same point.”*

**Corrected.**
 *“on west ...radiating to east.” -> “in the west ...radiating to the east.”*

**Corrected.**
 *“originating from west of the Atlantic forest ...northwest to Pampa”. This is not clear.*

*The only Pampa locality is south of all Atlantic forest collection sites.*

**The sentence was corrected to: “originating from the west of the Atlantic Forest… to the south in the Pampa”. Thank you for noting this mistake.**

*P5: “...did not recover none population ...” -> “...did not recover any population …”*

**Corrected.**
 *P6: “In fact, EBPS ...” -> “In fact, EBSP...”*
**Corrected.**
 *Environmental distribution patterns and ecological requirements
P2: “...overlaps at the Southern Brazil ...” -> “...overlaps in Southern Brazil ...”*

**Corrected.**
 *P3: “... in any of these cases the null hypothesis of niche equivalence could be rejected”. This is not what is stated in the Discussion, or what Table S15 shows. It should state that the null hypothesis of niche equivalency could not be rejected.*

**Corrected to: “the null hypothesis of niche equivalency could not be rejected in any pairwise comparison”.**

***Discussion:*** *P3 (and general comment about Wolbachia throughout the manuscript): The first sentence here (“Additionally, the congruence...”) needs more explanation. It is not clear what is meant by “selective scanning assoc. with three Wolbachia invasions”. If three different Wolbachia invasions fixed in the sufficiently distant past (your analyses do suggest relatively ancient lineage divergence), I’m not sure how your results would look any different. I guess I’m just not clear on what is being described here.
Only VERY recent Wolbachia invasions (i.e. not yet fixed in the invaded population) or maintenance of intermediate Wolbachia frequencies would remove a signal of reciprocal monophyly in the mtDNA. However, fixed Wolbachia invasions (anytime after lineage divergence) could produce discordant mtDNA vs. nucDNA topologies (in addition to significant reductions in tajima’s D in mtDNA loci, depending on timing), which your analyses show. A more general comment: Do these species have Wolbachia? I like that the Wolbachia is discussed as a potential factor that could disrupt species delimitations, but there is nothing presented as to whether Wolbachia even exists (or existed) in this clade. A simple PCR diagnostic on some of your samples is certainly warranted here. Or at the very least a few sentences describing what is known about the presence of Wolbachia (and their associated phenotypes, if known) in these or closely related species. All of this is to say that your primary conclusion is not affected (you still convincingly have three reciprocally monophyletic species) but I think there is certainly a possibility that Wolbachia (or other endosymbionts) could have influenced some of the phylogenetic and population genetic patterns observed.*

**This reasoning is now considered in P3 (lines 532-554): “In this study, besides the congruent results recovered by both mitochondrial markers (COI and COII), the nuclear markers AMD and HB also supported the reciprocal monophyly of three or at least two of the lineages, respectively. The use of both ABGD algorithm and coalescence-based Bayesian analysis, using single or multi-locus strategies, reinforced the subdivision of the M. projectans complex into at least three clusters, with an exact correspondence to the three lineages previously defined using DNA Barcoding. These results allowed rejecting the hypothesis that the clades observed by Machado et al. [25] would be a result of selective scanning associated with three different and recent invasions of Wolbachia in M. projectans [28]. Nevertheless, it is still possible that such patterns are resultant of ancient invasions independently fixed in different populations, that were first associated with cytoplasmic incompatibility, and post-zygotic isolation, and later enhanced the emergence or pre-zygotic isolation through reinforcement [91-93]. In this case, Wolbachia could act as a pro-speciation factor, and such a longstanding infection could result in fixation also for nuclear alleles. In fact, there is evidence for Wolbachia infection in at least one species of the Zygothrica genus group, and this endosymbiont was shown to be more frequent in mycophagous than non-mycophagous Diptera [29]. Although at a first sight simple diagnostic amplification could be used to confirm the presence of Wolbachia in our samples, shortages related to the amplification success presented by some of the markers employed here would not allow a confident description of prevalence patterns with such a methodology. Thus, it remains to be further evaluated if Wolbachia could or not be involved in the speciation of the M. projectans complex. Even so, this uncertainty does not affect the conclusion about the presence of at least three species under the general morphotype previously ascribed to M. projectans.”**

*P5: “... M. projectans affinis 2 presents higher suitability for this region [Dominican Republic].” This is very difficult to see in the figure. Is there a way to easily quantify this that shows higher suitability relative to the other species?*

**This was now tested with an ANOVA test, as stated at lines (574-577): “mean logistic suitability of 0.67, against 0.57 and 0.50 presented for M. projectans affinis 1 and M. projectans affinis 1, respectively (F = 4.74, p < 0.05, in an ANOVA performed with suitability values presented by each species across 25 random points chosen around the area”.**

*P8: “...seems to have been hold despite ...” -> “ ...seems to have been maintained despite ...”*

**Corrected.**

*” ...allows us hypothesizing ...” -> “ ...allows us to hypothesize ...”*

**Corrected.**

**Reviewer 3:**

**Reviewers Comments to the Author**

*I think the biological problem is interesting, but the sample size to answer the biological question is not significant. The sampled area is restricted to interior south Atlantic Forest and Pampa and COI mt sequences are predominant. I think the authors have data to elaborate hypotheses about diversification patterns for the group but not to discuss process.*

**This sampling size was only accomplished after several collections performed in different Brazilian municipalities. Specimens of the *M. projectans* complex were found in 45 localities, but sample sizes in some cases was reduced to a few individuals. Furthermore, only males were used due to shortcomings related to the effectiveness of the identification process (see above). When DNA was finally extracted, frequent amplification failures were faced, which commonly lead to DNA termination (see above). Thus, we really understand the reasoning of the reviewer, but we can ascertain that several different trials were performed to circumvent these biases. Our results were attained after an intense effort, and we think they provided several interesting clues about the diversity and the evolution of the complex, although it leaves some interesting open questions.**

**Reviewer 4:**

**Reviewers Comments to the Author**

*This paper examines the phylogeography of the Mycodrosophila projectans species complex, a mycophagous lineage in the Zygothrica Genus Group. The authors have thoroughly analyzed a combination of mitochondrial and nuclear gene data to discover three cryptic species in this complex. Overally, I found this a very easy paper to read and review. The authors did a wonderful job explaining all of their analyses. I only have a couple minor comments about the dating analyses.*

*→ How did you select the outgroup taxa for this study? This isn’t really justified in the paper and the species selected seem like a random mix of taxa across the phylogeny, with a slight focus on the Hawaiian Drosophila-Scaptomyza lineage. Was this done to facilitate dating analyses?*

**We are really grateful for your comments and suggestions. The selection of the outgroup taxa is now explained in lines (264-270): “additional sequences were downloaded from GenBank for D. melanogaster (Sophophora sub-genus), D. virilis (Siphlodora subgenus), D. ornatifrons (Drosophila subgenus), D. grimshawii and Scaptomyza (Hawaiian drosophilids) from the Drosophilinae subfamily, and P. variegata from Steganinae subfamily (see S2 Table). These species represent a set of different lineages within Drosophilidae phylogeny [78], and were employed as successive outgroups as well as to establish further calibration points within our phylogeny (see below).”**

*→ The Tamura and Survov dates are quite different from one another. Perhaps this might warrant some discussion? Obbard et al. (2012) do discuss a wide range of dates and it might be helpful to refer to this paper in your discussion.*

**The use of Tamura and Suvorov dates is now discussed, and the results of Obbard et al. (2012) are referred in the paper (see lines 611-625): “This divergence timing was established using a fossil calibration [79,80], and two contrasting molecular clock estimates provided by Tamura et al. [81] and Suvorov et al. [82] for the divergence between Sophophora and the remaining Drosophila. The first of these studies employed 176 nuclear genes to estimate a genomic mutation clock rate, using the time of formation of Kauai Island in Hawaii as a prior to the divergence of D. picticornis and other species of the planitibia group of Hawaiian drosophilids [97]. Suvorov et al. [82] employed 155 genome assemblies to generate a matrix of 2,791 genes, which was used to reconstruct a fossil calibrated phylogeny, based on at least five fossil datings. As biogegraphical calibrations can overestimate divergence timings, leading to underestimations of rates [98], whereas fossil calibrations can underestimate divergence timings, leading to overestimated rates [99], we preferred to use intermediate values to accommodate these biases. Interestingly, using these priors, we obtained a mean divergence time of 39.7 Mya [95% HPD Interval 24.5 – 55.4 Mya] which is quite similar to the results obtained by Obbard et al. [98] using a mutation rate establish for four-fold degenerate codons, which added further confidence to our results.”**
