# Supplementary material for: Unveiling the Mycodrosophila projectans (Diptera, Drosophilidae) species complex: Insights into the evolution of three Neotropical cryptic and syntopic species
Source: PLoS One. 2022 May 25;17(5):e0268657. doi: 10.1371/journal.pone.0268657 (PMC9132268; doi:10.1371/journal.pone.0268657)
Supplement: Attachment — Submitted filename: Myco_PlosOne_WJE.docx [file pone.0268657.s024.docx]

**Unveiling the *Mycodrosophila projectans* (Diptera, Drosophilidae) species complex: insights into the evolution of three Neotropical cryptic and syntopic species**

Stela Machado^1*^ [stelamchd@gmail.com](mailto:stelamchd@gmail.com)

Maiara Hartwig Bessa^1*^ [bessamai@gmail.com](mailto:bessamai@gmail.com)

Bruna Nornberg^2^ [brunanornberg@gmail.com](mailto:brunanornberg@gmail.com)

Marco Silva Gottschalk^3^ [gotts007@yahoo.com](mailto:gotts007@yahoo.com)

Lizandra Jaqueline Robe^1#^ [lizbiogen@gmail.com](mailto:lizbiogen@gmail.com)

^1^Universidade Federal de Santa Maria (UFSM), Programa de Pós Graduação em Biodiversidade Animal, Avenida Roraima 1000, CEP 97105-900 - Santa Maria, RS - Brazil, Phone: +55 55 3220-8912

^2^Universidade Federal do Rio Grande (FURG), Instituto de Ciências Biológicas – ICB, Av. Itália, km 08 Campus Carreiros, CEP 96203-900 - Rio Grande, RS – Brasil, Phone: +55 53 3293-5128

^3^Universidade Federal de Pelotas (UFPel), Instituto de Biologia, Departamento de Ecologia, Zoologia e Genética, Campus Capão do Leão, Mail Box 354, CEP 96010-900 - Capão do Leão, RS – Brazil, Phone: +55 53 3275-7340

* Both authors contributed equally to this work.

\# Corresponding Author

**Abstract**

The *Zygothrica* species group has been shown to be speciose with a high number of cryptic species. DNA Barcoding approaches have been a valuable tool to uncover cryptic diversity in this lineage, as recently suggested for the Neotropical *Mycodrosophila projectans* complex, which seems to comprise at least three different species. The aim of this study was to confirm the subdivision of the *M. projectans* complex, while shedding some light into the patterns and processes related to its diversification. In this sense, the use of single and multi-locus datasets under phylogenetic, distance, coalescence and diagnostic nucleotide approaches confirmed the presence of at least three species under the general morphotype previously described as *M. projectans*. Only a few subtle morphological differences were found for the three species for aedeagus morphology and abdominal color patterns that were described. Ecologically, sympatry and syntopy seems to be recurrent patterns for these three cryptic species, involving widely overlapping niches, implying niche conservatism. This morphological and ecological similarity has persisted though cladogenesis within the complex beginning in the Miocene, providing an interesting example of morphological conservation despite ancient divergence. These results, in addition to contrasting patterns of past demographic fluctuations, allowed us to hypothesize patterns of allopatric or parapatric diversification, leading to secondary contact with expansion within their current geographical ranges. Even so, genetic diversity was generally high within species, suggesting migration may encompass an adaptive response to the restrictions imposed by the ephemerality of resources.

**Keywords:** phylogeography, Quaternary, Pleistocene, mycophagous, environmental niche modeling

**Introduction**

The Neotropical region harbors around 13% of the world’s biodiversity [1, 2] encompassing five megadiverse countries [3] and three major biodiversity hotspots [4]. Although the processes responsible for the origin of this diversity have been previously discussed [5-7], it is still estimated that for each known Neotropical species, ten remain unknown [1]. Molecular taxonomy, and in particular the DNA Barcoding approach, have contributed significantly to the task of unraveling these high levels of unknown or cryptic diversity [8]. These tools are especially important when speciation occurs in the absence of morphological divergence, a phenomenon known as cryptic speciation [9-12]. The mechanisms responsible for this pattern are not well understood [13], but it has been suggested that sexual selection acting at the level of non-visual signals [14] may be responsible.

The family Drosophilidae includes more than 4,300 species found in almost all biogeographic regions of the planet [15-17]. Different species are associated with diverse resources like fruits, fungus, sap, pollen and rotten leaves [16, 18]. This ecological diversity is largely due to the saprophagous nature of drosophilids that feed mainly on bacteria and yeast involved in fermentation and on fermentation by-products [15, 16, 19]. Nevertheless, many species are also associated with macroscopic fungi, using them as a resource for feeding (eating spores and hyphae [20]), oviposition and/or breeding sites. This is the case for the *Zygothrica* species group which encompasses five mycophagous genera: *Hirtodrosophila*, *Mycodrosophila*, *Paraliodrosophila*, *Paramycodrosophila* and *Zygothrica*.

*Mycodrosophila* Oldenberg, 1914 comprises around 130 obligatory mycophagous species, which depend on fungi in all stages of their life cycle [21]. This genus seems to have originated in the Old World, later migrating to the New World, where it currently occupies the Nearctic and Neotropical regions [15, 17, 22]. Although only 4% of its diversity has so far been reported in the Neotropics [17, 21], there is evidence suggesting the diversity of *Mycodrosophila* in this biogeographic region might be underestimated due to sampling bias [23]. In Brazil, for instance, the number of species described in this genus has nearly doubled in the last two years, due to integrated taxonomical efforts, implementation of traditional morphological techniques, and DNA Barcoding [22, 24, 25]. The latter approach suggested the existence of entirely cryptic species of *Mycodrosophila*, which cannot be easily differentiated even at the level of male genitalia [25].

*Mycodrosophila projectans* (Sturtevant, 1916) has been considered a widely distributed Neotropical species, with records from Mexico to Brazil [22, 26]. However, when some of these Neotropical populations were evaluated through DNA Barcoding approaches using only nucleotide sequences of the mitochondrial cytochrome oxidase c subunit I (COI) gene, at least three different lineages were uncovered [25]: *M. projectans*, *M. projectans* affinis 1 and *M. projectans* affinis 2. However, these results should be interpreted with caution because the sole use of mitochondrial genes may be misleading due to the presence of nuclear paralogues of mitochondrial genes (numts [27]), or “hitchhiking” with maternally inherited endoparasites [28]. The presence of numts can lead to divergent sequences even within the same genome [27], and the latter can modify the matrilineal history of mitochondrial genes both from intraspecific or interspecific populations, causing homogenization or divergence at the level of their mitochondrial nucleotide sequences [28]. The endoparasite *Wolbachia* has already been reported in some groups of mycophagous drosophilids, including one species of *Hirtodrosophila*, so mushrooms may be hotspots for horizontal transfer of this endoparasite where multiple drosophilid species co-exist [29]. Including nuclear gene sequences or morphological markers along with mitochondrial genes may allow insight into the evolutionary relationships.

The aim of the present study was to confirm the subdivision of the *M. projectans* complex, while shedding some light into the patterns and processes related to its diversification. We increased sampling for the mitochondrial COI gene, incorporated sequences of the mitochondrial cytochrome oxidase c subunit II (COII) gene and nuclear hunchback (HB) and alpha methyldopa (AMD) genes. We also performed further morphological and ecological niche modeling evaluations. These analyses allowed us to confirm the existence of at least three species within the *M. projectans* complex and to indicate some putative autapomorphies related to abdominal color and male genitalia patterns. Morphological and ecological similarity of the three species was notable, especially in the face of divergence dated back to the Miocene.

**Materials and methods**

**Sampling**

Collections were made in 45 sites distributed along Amazonian, Atlantic Forest, and Pampa Brazilian Biomes (S1 Table, Fig 1). The Amazonian biome occupies around 6.7 million km^2^, and its vegetation includes dry land forests, floodplain forests and *igapós* [30]. The Atlantic Forest includes ca 1.1 million km^2^ along coastal Brazil with a large latitudinal range encompassing tropical and subtropical regions [31]. The Pampa biome is restricted to the state of Rio Grande do Sul and occupies an area of 176.5 km^2^, characterized by grasslands with sparse shrub and forests within the South Temperate Zone [32]. In these localities, active searches for fruiting bodies of macroscopic fungi were performed in forest fragments during the active period of the flies. Adult adults resting or flying over the fungi were collected with an aspirator [33] and, in some cases, fungi were returned to the laboratory and maintained until any adults emerged. Flies were stored in absolute ethanol and an initial identification was performed based on external morphology and male genitalia. Only males were used because morphological differences and aedeagus differences reliably confirmed species identification. All material was collected in accordance with Brazilian law, under scientific collection license (SISBIO number 28013-12). As practices did not involve vertebrate species, Brazilian law does not require approval in Ethics Committee on Animal use.

**Amplification, sequencing and manipulation of molecular markers**

Total DNA of each adult male was extracted following the phenol-chloroform protocol described by Sassi et al. [34] or using the NucleoSpin Tissue XS kit (Marchery-Nagel). Mitochondrial COI genes were amplified using different combinations of the primers TYJ1460 and C1N2329M (5’ACTGTAAATATATGATGAGCTCATACA3’), modified from Simon *et al.* [35], HCO1490 and LCO2198 [36] or COIMYCOF (5’AYTTTATTTTYGGRGCHTGR3’) and COIMYCOR (5’WCCTAATGADCCAAADGTTTCY3’), whereas the mitochondrial COII gene was amplified using primers TL2J3037 and TKN3785 [35] or COII3494M (5’GGNARVAYDRYDCGRTTRTCDAC3’) and COII3400M (5’ATYGGNCAYCARTGRTAYTGA3’), modified from Simon et al. [35]. The nuclear HB gene was amplified with primers HB106F and HB903R [37] or HBFMYCOS (5’CATATACGCAAGCACAAGAACC3’) and HBRMYCOS (5’GCTCRGCACTGGCMGCAC3’), whereas the AMD gene was amplified with primers AMDEX4F [38] and AMDBW [39]. The amplicons obtained were purifying using a solution of polyethylene glycol (PEG) 13% and NaCl 1.6M. Sanger sequencing was performed by Macrogen (http://www.macrogen.com/eng/).

Resulting electropherograms were assembled using the Staden Package Gap 4 program [40], where each contig was individually checked regarding sequence quality and editing for polymorphic sites used the IUPAC degeneracy code. Orthologous sequences were aligned with the Clustal W algorithm as implemented in Mega X [41]. Phase 2.1 [42, 43] was used to determine the gametic phase of the nuclear sequences, as implemented in DNAsp 6 [44]. Mitochondrial datasets were checked for the presence of stop-codons and frameshifts to avoid the presence of numts. The ratio of the number of nonsynonymous substitutions per nonsynonymous sites (dN) and the number of synonymous substitutions per synonymous sites (dS) was assessed in Mega X using the Kimura 2-Parameters nucleotide substitution model (K2P) [45]. The same method was also used to perform a codon-based-Z test of purifying selection (for details see [25, 46]).

**Morphological and molecular approaches for species delimitation**

As Machado *et al.* [25] employed a DNA Barcoding approach suggestin *M. projectans* was a potential species complex, both morphological and molecular approaches were used here to delimit species. First, adult males were photographed with a stereoscopic microscope to determine abdominal color patterns and each right wing was removed for further morphometric analysis (see below). Second, using confocal microscopy, we assessed differences in male aedeagus morphology based on Wheeler & Takada [22]. Male terminalia were treated with 10% potassium hydroxide (KOH) and 70% ethanol, following Klaus et al. [47], and dissected in glycerol. Visualization was performed on a Leica TCS SP8 scanning spectral confocal microscope. Images were acquired using a 20x objective lens, with a 488nm laser adjusted to full power (11.6mW) and emission signals detected under 500 to 584nm.

All individuals were identified by DNA Barcoding in Bold [53] using COI sequences. Phylogenetic analyses were then performed for each individual gene based on Bayesian inference (BI), to assess phylogenetic structure as well as reciprocal monophyly of each species. In each case, the best codon partitioning schemes as well as their respective best substitution models were determined by the Akaike Information Criterion (AIC) [54], as performed in Partition Finder 2.1.1 [55]. The BIs were performed in MrBayes 3.2 [56], with at least 50.000.000 generations, sampling every 10.000 and burning 25% of the initial trees. Each analysis was ended after the average standard deviation between two independent runs was below 0.01, which assures convergence of the results. These analyses were performed using *Mycodrosophila elegans*, MycoNova_SFP_403B1, a sequence characterized by Machado et al. [25] for an undescribed Neotropical species of *Mycodrosophila*, and *D. melanogaster* as outgroups. Sequences for these taxa were downloaded from GenBank (see S2 Table).

Two species delimitation tests based on coalescence approaches that do not require *a priori* species allocation were also employed: the Generalized Mixed Yule Coalescent method (GMYC) [57, 58], which was designed to find genetic clusters evolving independently using single locus datasets, and a multi-locus species delimitation method implemented in the Stacey package [59]. In both cases, substitution models were selected according to the AIC, as performed in jModelTest 2.1.10 [60, 61] employing datasets comprising sequences of the *M. projectans* complex, plus sequences previously generated by Machado et al. [25] for *Mycodrosophila elegans* and MycoNova_SFP_403B1 (S2 Table). In the first case, ultrametric trees were reconstructed in Beast 2.6 [62], with MCMC encompassing at least 50,000,000 iterations sampling every 1,000-5,000 chains and burning in the first 10%. Further evaluations of the trees were performed using a single threshold analysis in the Splits package (https://github.com/tfujisawa/splits_tmp) in R 1.3.1093 [63], which detects transitions between inter and intra-specific processes. Meanwhile, Stacey analyses was performed in Beast 2.4.8 [64] under unlinked substitution models, unlinked relaxed molecular clock models and unlinked Stacey coalescent parameters, with ploidy level established according to the respective marker. The MCMC encompassed 200,000,000 generations, saving every 10,000, and burning in the first 10%. Convergence was evaluated in Tracer 1.60 [65], checking likelihood and posterior probability traces and confirming that all ESS values are above 200. Clustering schemes and species assignments were later evaluated using Species Delimitator Analyzer [66], with a collapse height value of 0.0001.

Additionally, the Automatic Barcode Gap Discovery (ABGD) algorithm was applied for each COI, COII, COI+COII, HB and AMD datasets, under the Kimura 2-Parameters substitution model, as performed on the website https://bioinfo.mnhn.fr/abi/public/abgd/abgdweb.html. The ABGD groups individuals based on the pairwise distance observed between sequences, without *a priori* hypothesis [67]. Finally, the assignments obtained by the strategies described above were employed to measure intra- and interspecific nucleotide distances and to obtain diagnostic characters for each species of the individual datasets (COI, COII, COI+COII, HB and AMD datasets). Pairwise distances were calculated on MEGA X using the Kimura 2-parameters nucleotide substitution model. Diagnostic nucleotides were recovered for each putative species using the Spider package [68] in R.

**Morphometric analysis**

The right wings of males of the *M. projectans* complex were photographed with a SteREO DiscoveryV20 microscope, with the program AxioVs40 V 4.8.1.0 (Carl Zeiss Imaging Solutions GmbH), with an increase of 37.5X. TPSdig2 [69] was used to mark ten landmarks chosen based on wing venation, representing bifurcations, intersections and tip veins (S1 Fig, S3 Table). Partial and relative warps, centroid size and a weight matrix were then calculated with TPSRelwn [70], where a graphic representation from shape variation was obtained. Analysis of variance (ANOVA) was performed to compare the centroid size of the wings within and among species, and a canonical variate analysis (CVA) was performed to compare their relative warps. ANOVA and CVA were performed in PAST 3 [71].

**Diversity and population structure analyses**

Diversity estimates [number of haplotypes (H) or alleles (A), haplotype diversity (Hd) or expected Heterozigosity (He), nucleotide diversity (π), and number of polymorphic sites (*s*)] and neutrality tests [Tajima’s D [72], Fu’s and Li’s D and Fu’s and Li’s F [73]] were performed in DNAsp 5.10.01 [74]. Genealogical relationships between haplotypes or alleles (COI, COI+COII, AMD and HB) were estimated by median-joining, as implemented in Network 10.1.0 [75].

Pairwise fixation indexes (Fst) among species and populations were estimated for each dataset in Arlequin 3.5 [76], with 1,000 permutations. Analysis of Molecular Variance (AMOVA) was also performed with the same software subdividing sequences by species (as identified by DNA Barcoding) and populations (according to sampling localities), to assess the covariance among groups or species (Φct), among populations within groups or species (Φsc) and the covariance within populations (Φst). A Mantel test was performed in Arlequin 3.5 to access the correlation between geographical distances and genetic divergences. Finally, a Bayesian analysis of population structure (BAPS) was performed for mitochondrial markers using BAPS 6.0 [77], to investigate population subdivision without *a priori* assignments. These two last analyses were performed only for COI, COII and COI+COII datasets.

**Dating**

A chronophylogenetic analysis was performed in Beast 2.6.3 [64] under a multi-species coalecent and a relaxed molecular clock approach, using the StarBeast package to construct a putative species tree based on the gene trees estimated for COI, COII, HB and Amd. For this task, besides the new sequences characterized here for the *M. projectans* complex, additional sequences were downloaded from GenBank for *D. melanogaster* (*Sophophora* subgenus), *D. virilis* (*Siphlodora* subgenus), *D. ornatifrons* (*Drosophila* subgenus), *D. grimshawii* and *Scaptomyza* (Hawaiian drosophilids) from the Drosophilinae subfamily, and *P. variegata* from Steganinae subfamily (see S2 Table). These species represent a set of different lineages within the Drosophilidae [78], and were employed as successive outgroups as well as to establish further calibration points within our phylogeny (see below). Unlinked molecular substitution models were employed for each gene according to the results provided by the AIC performed in jModelTest. Unlinked substitution rates were estimated for each gene based on calibrations to the split between *Scaptomyza* and *D. grimshawii* using a prior with a mean of 17.5 Mya and stdev of 1.25 Mya, according to the fossil data [79, 80] and to the divergence between *Sophophora* and the remaining *Drosophila* with a prior of a mean of 55 Mya and stdev of 8 Mya, according to the average calculated between the molecular clock dating estimated by Tamura et al. [81] (~62.9 Mya) and Suvorov et al. [82] (~47 Mya)]. Unlinked gene trees were also evaluated under a Yule model, with ploidy levels established according to the inheritance pattern for each marker. The analysis was performed for 800 million iterations, storing every 10,000, and discarding the first 10% as burn in. The run was then visualized in Tracer 1.6 to assure convergence and adequate sampling. Results were summarized in TreeAnnotator 1.8.0 [83] and visualized in FigTree 1.4.3 [84].

Extended Bayesian skyline (EBPS) [64] analyses were also performed for each species in Beast 2.6.3 employing COI and COII datasets. This analysis was performed using unlinked substitution models, as selected by the AIC performed in jModelTest. Priors for the unlinked relaxed molecular clock models were established based on the mean of the ucld.means obtained for each gene by the chronophylogenetic analysis described above. Unlinked extended Bayesian models were finally applied for each marker, with populations models assigned according to differences in effective population sizes. Each analysis encompassed at least 500 million iterations, sampling every 10,000, and burn in of the first 10%. These results were evaluated in Tracer 1.6 and skyline plots were constructed in R.

**Species distribution modeling**

Potential distribution maps were constructed for each species of the *M. projectans* complex based on environmental niche modeling (ENM) [85]. Bioclimatic variables were first downloaded from the Paleoclim database [86] at a resolution of 2.5 mins, and then cut with a shapefile encompassing the Neotropics. Pearson correlations between the cut layers were estimated in R using the packages Raster [87] and corrplot [88], and the seven least correlated abiotic variables were employed in the modeling strategy: temperature seasonality (Bio-4), mean temperature of warmest quarter (Bio-10), mean temperature of coldest quarter (Bio-11) precipitation of the wettest quarter (Bio-16), precipitation of the driest quarter (Bio-17), precipitation of the warmest quarter (Bio-18) and precipitation of the coldest quarter (Bio-19). As cryptic diversity in the *M. projectans* species complex was previously neglected, only the sampling points recorded in this study (S1 Table) were used. To decrease the effect of sampling bias, background points were selected along a radius of 500 km of each sampling point.

Models were generated in R 1.3.1093 using the maximum entropy algorithm function contained in the “dismo” package [89], with calibration to the present and projection to four moments of the past: Late Pliocene (3.3 Mya), the Marine Isotope Stage 19 (MIS19) in Pleistocene (MIS, *c*. 787 Kya), Last Interglacial (LIG, *c.* 130 kya), and Last Glacial Maximum (LGM, *c.* 21 kya). In each case, 75% and 25% of the registers were employed as training and test, respectively, in 25 cross validation replicates. The accuracy of the predictive distribution models was accessed through the Area Under the Receiving Operating Curve (AUC) mean value.

Measurements of abiotic niche overlap between species and identity tests were performed in R using the ENMTools package [90]. To this task, pairwise niche overlap was evaluated according to Schoener’s D and Hellinger’s I, for which 0 means no overlap and 1 indicates that niches are identical. To identify if observed measures were significantly different from the expectations drawn from the null hypothesis of niche equivalency, the null distribution was derived from 25 replicates, in each of which registers were pooled and randomized and the potential distribution maps were derived under the maximum entropy algorithm.

**Results**

A total of 172 individuals first recognized as *M. projectans* were collected and the following numbers of sequences were characterized: 172 for COI (~727bp), 70 for COII (~560bp), 20 for AMD (~921bp) and 25 for HB (~398bp) (Table 1; for accession numbers, see S2 Table). Sampling disparity occurred due to frequent failures of amplification that commonly led to the premature termination of some DNA samples. No sequences contained stop codons or frameshifts and all mitochondrial sequences that did not reject the null hypothesis of neutrality in codon-based-Z test of purifying selection and showed a dn/ds ratio near one showed low K2P distances (d < 1.3%), suggesting stochastic deviation in intraspecific variation (S4 Table).

Table 1. Summary information regarding sample size (N), divergence (MI%, MX% and MD%) and diversity analyses (π%, H or A, Hd or He and S) and results of neutrality tests (Tajima’s D, Fu and Li’s D and Fu and Li’s F) performed for each of the three species of the *M. projectans* complex with each of the employed mitochondrial and nuclear markers.

|  |  | **Species** | **N** | **MI%** | **MX%** | **MD% (SD%)** | **π%(SD%)** | **H or A** | **Hd or He** | **S** | **Tajima's D** | **Fu and Li's D** | **Fu and Li's F** |
| --- | --- | --- | --- | --- | --- | --- | --- | --- | --- | --- | --- | --- | --- |
| mtDNA | COI | *M. projectans* aff. 1 | 33 | 12.1 | 4.8 | 0.4 (0.1) | 0.3 (0.2) | 7 | 0.383 | 20 | ***-2.32*** | ***-3.52*** | ***-3.69*** |
|  |  | *M. projectans aff. 2* | 61 | 12.3 | 3.1 | 0.8 (0.1) | 0.7 (0.1) | 37 | 0.931 | 51 | **-2.04** | **-3.12** | ***-3.24*** |
|  |  | *M. projectans aff. 3* | 78 | 12.1 | 6.1 | 1.5 (0.3) | 1.3 (0.1) | 38 | 0.932 | 49 | -1.29 | ***-3.63*** | ***-3.25*** |
|  | COII | *M. projectans aff. 1* | 9 | 9.7 | 1.8 | 0.4 (0.5) | 0.4 (0.2) | 3 | 0.417 | 10 | **-1.84** | ***-2.09*** | **-2.27** |
|  |  | *M. projectans aff. 2* | 22 | 9.7 | 2.4 | 0.8 (0.5) | 0.8 (0.1) | 14 | 0.896 | 22 | -1.05 | -1.19 | -1.34 |
|  |  | *M. projectans aff. 3* | 39 | 11.4 | 2.7 | 1.4 (0.7) | 1.2 (0.1) | 27 | 0.974 | 34 | -0.75 | -2.05 | -1.90 |
|  | COI + COII | *M. projectans aff. 1* | 9 | 12.5 | 1 | 0.4 (0.1) | 0.3 (0.1) | 5 | 0.722 | 16 | ***-1.92*** | ***-2.18*** | ***-2.37*** |
|  |  | *M. projectans aff. 2* | 18 | 12.5 | 1.7 | 0.8 (0.1) | 0.7 (0.08) | 14 | 0.954 | 38 | -0.97 | -1.33 | -1.42 |
|  |  | *M. projectans aff. 3* | 39 | 12.5 | 2.8 | 1.4 (0.2) | 1.4 (0.05) | 30 | 0.978 | 72 | -0.43 | -1.82 | -1.58 |
| nDNA | AMD | *M. projectans aff. 1* | 3* | 4.2 | 0.3 | 0.2 (0.1) | 0.2 (0.04) | 3 | 0.8 | 4 | 1.18 | 1.47 | 1.49 |
|  |  | *M. projectans aff. 2* | 9* | 4.2 | 2.3 | 1.2 (0.6) | 1.2 (0.1) | 9 | 0.941 | 34 | 0.34 | ***1.66*** | 1.48 |
|  |  | *M. projectans aff. 3* | 8* | 4.3 | 7 | 2.9 (2.5) | 2.8 (0.7) | 12 | 0.967 | 76 | -1.46 | -0.43 | -0.73 |
|  | HB | *M. projectans aff. 1* | 6* | 3.5 | 1.3 | 0.4 (0.4) | 0.4 (0.1) | 5 | 0.667 | 4 | -1.02 | -0.46 | -0.68 |
|  |  | *M. projectans aff. 2* | 5* | 2.5 | 2.2 | 0.9 (0.5) | 0.9 (0.2) | 7 | 0.911 | 7 | -0.77 | -0.13 | -0.32 |
|  |  | *M. projectans aff. 3* | 14* | 2.5 | 2.6 | 0.6 (0.5) | 0.6 (0.1) | 10 | 0.704 | 13 | **-2.01** | **-2.82** | **-3.01** |

N = number of specimens, MI% minimum interspecific distance in percentage, MX% maximum intraspecific distance in percentage, MD% mean intraspecific distance in percentage with standard error, π nucleotide diversity, H number of haplotypes (for mitochondrial markers), A number of alleles (for nuclear markers), Hd haplotype diversity (for mitochondrial markers), He expected heterozygosity (for nuclear markers), S variable sites. In bold significance of p < 0.05 and in bold and italic p < 0.02. *The number of alleles analyzed for nuclear markers was doubled after PHASE analysis.

**How many *M. projectans* species are there ?**

**Molecular differentiation**

~~According to Bold System~~, Of the 172 COI sequences subdivided among the three species already reported for the *M. projectans* complex by Machado et al. [25], 33 were identified as belonging to *M. projectans* affinis 1, 61 to *M. projectans* affinis 2 and 78 to *M. projectans*, hereafter referred as *M. projectans affinis* 3. All individuals were recovered as a highly supported monophyletic lineage in the BI performed with COI (PP=0.93), COII and Amd (PP = 1.00); whereas for HB, individuals of *M. projectans* affinis 1 were recovered as a basal grade (PP ≤ 0.68) (S2 Fig). With the exception of this grade recovered for *M. projectans* affinis 1 with HB, the three species were revealed to be reciprocally monophyletic (PP ≥ 0.99). The analyses performed with GMYC also resulted in three clusters for COI, although COII and Hb further subdivided *M. projectans* affinis 3 into three and two clusters, respectively, and AMD joined *M. projectans* affinis 1 and *M. projectans* affinis 3 in one cluster (S5 Table). Even so, the delimitation with higher PP recovered in the multi-locus coalescent Bayesian-based analysis executed in Stacey supported the subdivision of the *M. projectans* complex in three clusters (PP=0.015) (S5 Table), in accordance with the separation seen in the phylogenetic analyses. Finally, the ABGD analysis also resulted in three clusters for COI, COII, COI+COII and HB datasets, although AMD defined only two groups of sequences, one of which grouped sequences of *M. projectans* affinis 1 and *M. projectans* affinis 3 (S5 Table).

Considering the delimitation in three species for COI, *M. projectans* affinis 1, *M. projectans* affinis 2 and *M. projectans* affinis 3*,* showed maximum intraspecific distances of 4.8%, 3.1% and 6.1%, respectively (Table 1). The minimum value from COI interspecific distances was 12.1%, as recovered in a comparison involving individuals of *M. projectans* affinis 1 and *M. projectans* affinis 3. The analysis of diagnostic characters performed for COI resulted in 18 diagnostic sites for *M. projectans* affinis 1, 17 for *M. projectans* affinis 2 and 11 for *M. projectans* affinis 3 (S6 Table). Generally, this pattern was repeated for COII, which showed a Barcoding Gap of 7.3% for the complex (maximum intraspecific distances of 2.7% and minimum interspecific distances of 10%) (Table 1) and recovered at least 21 diagnostic sites when the subdivision in three species was considered (S6 Table).

**Morphological differentiation**

Individuals of the *M. projectans* complex were extremely similar regarding external morphology, as we could identify three different abdominal color patterns. Two patterns were shared between *M. projectans* affinis 1 (n=12) and *M. projectans* affinis 3 (n=30), where the yellow spots of tergite IV were totally or partially separated by a posterior projection of the black area, hereafter called abdominal pattern A (Fig 2A and B; S7 Table). One pattern exclusive to *M. projectans* affinis 2 (n=24) was where tergite IV showed a black background and two yellow spots fully confluent in the dorsal region, hereafter called abdominal pattern B (Fig 2C; S7 Table). There were two exceptions to this pattern found in this species. A thorough examination of the description of *M. projectans* provided by Sturtevant [90], the redescription of Wheeler and Takada [22] and the holotype photos provided by the National Museum of Natural History (Washington D.C., USA) confirmed close resemblance of *M. projectans* affinis 2 with the described species.

Wing morphometry was used as a potential morphological marker in species identification for 57 individuals, of which 13 belong to *M. projectans* affinis 1, 19 to *M. projectans* affinis 2 and 23 to *M. projectans affinis 3.* The ANOVA and CVA tests did not show any statistically significant differences among species regarding the centroid size of wings and the scores of the relative warps, respectively (S3 Fig; S8 Table). In fact, a wide overlap of wing shape was detected among species (S3 Fig), so wing morpholoy did not provide enough resolution to distinguish species within the *M. projectans* complex.

At the male genitalia level, two, nine and six of *M. projectans* affinis 1, *M. projectans* affinis 2 and *M. projectans* affinis 3, respectively, were examined by optical microscopy, and seven, 14 and five by confocal microscopy, respectively. These evaluations revealed the presence of two male genitalia patterns within the *M. projectans* complex (Fig 3; S7 Table): one type of aedeagus structure was shared between *M. projectans* affinis 2 and *M. projectans* affinis 3 that resembles the illustrations provided by Wheeler & Takada ([22], pg. 396, figs. 15-18) for *M. projectans* (hereafter called type I) (Fig 3A); and a second type observed only in *M. projectans* affinis 1, with the aedeagus longer and with an apical region more rounded and wider in lateral view, as compared to the previous morphology (hereafter called type II) (Fig 3B).

**How and when did this diversification happen?**

**Phylogenetic relationships and divergence dating**

The resulting phylogenetic gene trees supported the monophyly of the *M. projectans* complex (PP = 1), although the topology of the relationships within the complex varied among genes. In fact, the mitochondrial markers recovered different topologies, with COI recovering *M. projectans* affinis 1 and *M. projectans* affinis 3 as a sister species (PP=0.33), where COII clustered *M. projectans* affinis 1 with *M. projectans* affinis 2 (PP=1). Conversely, both nuclear markers supported the clustering of *M. projectans* affinis 2 and *M. projectans* affinis 3 [PP=0.94 (HB) and PP=0.75 (AMD)] (Figs 4A-D). This incongruence is reflected in the low support reported for the early offshoot of *M. projectans* affinis 3 in the species tree (PP=0.50) (Fig 4E). According to this chronophylogenetic tree, the first divergence within the complex occurred around 17 Mya, where the next split occurred ca. 15 Mya.

**Patterns of genetic diversity and abiotic niches distinguish the three species and suggests modes of speciation**

**Genetic diversity, population structure and demographic history**

In general, the analysis of haplotype diversity (Hd) or expected heterozygosity (He) for mitochondrial and nuclear markers, respectively, nucleotide diversity (π) and number of polymorphic sites (S) revealed higher values for all four genes for *M. projectans* affinis 2 and *M. projectans* affinis 3 than for *M. projectans* affinis 1 (Table 1). Neutrality tests showed some significantly negative values for COI, COII, COI+COII and HB in all three species (Table 1).

Pairwise Fst indexes revealed significant values in the comparisons between species for all datasets, although the values recovered for AMD were lower than those reported for COI, COII, COI+COII and HB (S9 A-D Tables). In fact, AMOVA tests executed with the same sequences proved that at least 51% of the total variation was explained by differences among species (S10 Table). Conversely, the variation encountered among populations within the same species was very similar or even lower than that encountered within populations, especially for the nuclear markers (S10 Table).

The networks recovered for the three species using COI sequences generally showed star-like patterns, with central and more frequent haplotypes connected with peripheral and exclusive ones through few mutational steps (Fig 1). However, there were some exceptions, like haplotype 6 (Foz do Iguaçu/PR) in *M. projectans* affinis 1, haplotypes 4 (Teodoro Sampaio/SP) and 26 (Foz do Iguaçu/PR) in *M. projectans* affinis 2, and haplotypes 20 (Derrubadas/RS) and 29 (Pelotas/RS) in *M. projectans* affinis 3*,* that are separated by up to 7-17 mutational steps from their most similar haplotypes.

Although differentiation among species attained a minimum of 34 mutational steps for COI, intraspecific gene flow seems to have been maintained despite long distances. Thus, in *M. projectans* affinis 2, for example, haplotypes that were collected about 3,000 Km apart, like haplotype 37 and haplotype 7, were differentiated by only two mutational steps (Fig 1C). Likewise, in the same species, haplotypes differing by as much as 10 mutational steps were found at the same point. Notwithstanding, for *M. projectans* affinis 3, two haplogroups could be observed (Fig 1D); the first, with the most common haplotypes sampled in the west (H2 and H24) and radiating to the east, was almost restricted to Atlantic forest [only one individual found in the border between Atlantic forest and Pampa (at Santa Maria) shared a haplotype from this haplogroup), and the other almost entirely restricted to the Pampa biome, with several haplotypes being sampled along the border between the Atlantic forest and Pampa and radiating to the south. Conversely, the COI networks of *M. projectans* affinis 1 and 2 (Figs 1B and C) contained the most common haplotype originating from the west of the Atlantic Forest, from which it radiated haplotypes to the east in *M. projectans* affinis 1 and to east in the Atlantic Forest, to the south in the Pampa and to the north in the Amazon in *M. projectans* affins 2. These patterns were somewhat diluted in the COI+COII concatenated, AMD and HB networks, due to reduced sampling, although differentiation among species remained very pronounced (S4-S5 Figs).

According to BAPS, whereas COI did not recover any population subdivision, in any of the three species, COII subdivided *M. projectans* affinis 1 and *M. projectans* affinis 3 in two subpopulations and *M. projectans* affinis 2 in three subpopulations from two groups composed by individuals from the Atlantic Forest and another group exclusively composed by specimens from the Pampa biome (S6 Fig). Furthermore, the results provided by the Mantel Tests (S11 Table) revealed significant correlations between genetic and geographic distances only for *M. projectans* affinis 1 (rCOI=0.770, p=0.035) and *M. projectans* affinis 3 (rCOI = 0.398, p = 0.002; rCOII = 0.437, p = 0.026; rCOI+COII = 0.424, p = 0.029).

Results of the Extended Bayesian skyline (EBSP) analysis agreed with the neutrality tests and the star-like patterns seen for the COI networks. In fact, EBSP suggests *M. projectans* affinis 2 and *M. projectans* affinis 3 experienced an expansion of population size, but in different magnitudes and moments, initiated around 200 and 400 Kya, respectively (S7 Fig). According to the 95% HPD sum(indicators.alltrees) parameter, the number of effective population size changes in *M. projectans* affinis 3 and *M. projectans* affinis 2 ranged between 1 and 3 (median 1) and 1 and 3 (median 2), respectively. Nevertheless, for *M. projectans* affinis 1, as 0 was within the 95% HPD range of the sum(indicators.alltrees) parameter, EBSP did not provide significant evidence of changes in effective population size.

**Environmental distribution patterns and ecological requirements**

Currently, the three species of the *M. projectans* complex are sympatric in an area encompassing the southern region of Brazil (Fig 1, S1 Table), but *M. projectans* has been collected in tropical and subtropical areas from the Neotropical Region. In the areas sampled, species of the complex were found associated with fungal genera *Ganoderma*, *Polyporus* and *Trametes* (S12 Table), and at least twice all three species were found in syntopy. In these cases, the triad was simultaneously collected using *Ganoderma* and another unidentified fungus, at Derrubadas and Foz do Iguaçu, respectively, two conserved areas of Atlantic Forest. *Mycodrosophila projectans* affinis 2 and *M. projectans* affinis 3 also co-occurred in the genera *Ganoderma* and *Polyporus* at Bossoroca and Derrubadas, respectively (S12 Table).

ENM for all three species showed AUC values higher than 0.809 (S13 Table) suggesting the range of suitable areas differs among the three species, with present day overlapping ranges in Southern Brazil and possibly at different times in the past (Fig 5). Nevertheless, suitability of the sampling area seems to have decreased progressively during the Pleistocene, reaching the lowest levels during the LGM for all the three species.

Although the climatic variable that contributed most to the models in *M. projectans* affinis 2 and *M. projectans* affinis 3 was the precipitation of the driest quarter (Bio-17), for *M. projectans* affinis 1, the precipitation of the coldest quarter (Bio-19) was the most important variable (S14 Table). Measures of abiotic niche overlap based on ENM results were higher in the comparisons involving *M. projectans* affinis 1 and *M. projectans* affinis 2 (D = 0.904, I = 0.994), than when any of these were compared to *M. projectans* affinis 3 (D = 0.549, I = 0.839; D = 0.528, I = 0.858 respectively) (S15 Table). Thus, the null hypothesis of niche equivalency could not be rejected in any of the pairwise comparisons.

**Discussion**

This study revealed an additional example of cryptic diversity for *Mycodrosophila,* that was already hypothesized as having a number of cryptic species [25, 91]. Although the application of DNA Barcoding was fundamental to disentangling the *M. projectans* species complex, caution is necessary when interpreting results obtained solely with COI, since mitochondrial markers may suffer from the interference of numts or even hitchhiking due to maternally inherited endoparasites [28]. The influence of both of these factors on the proposed inclusion of three species in the *M. projectans* complex [25] can be discounted by the of evidence presented in the current study.

Concerns related to the co-amplification of numts were diminished by the absence of stop-codons, frameshifts, or high levels of divergence for COI sequences, patterns associated with neutral evolution that are commonly observed in paralogous copies of mitochondrial genes [38]. In addition, as numts generally cover a small portion of the mitochondrial genome [27], the congruence between the results obtained for COI and COII can be considered additional evidence against the presence of such artifacts here. In fact, for both COI and COII, we showed that reciprocal monophyly weas consistent with the presence of a gap between the maximum intraspecific distances and the minimum interspecific divergences. In addition, each of the tree species presented at least eleven diagnostic characters that seem to be fixed for each mitochondrial marker. Finally, for both markers, AMOVA indicated that more than 80% of the variation occurred among the three haplogroups, whose pairwise Fst values were close to 1.0.

In addition to the congruent results recovered COI and COII, the nuclear markers AMD and HB also supported the reciprocal monophyly of three or at least two of the lineages, respectively. The use of both the ABGD algorithm and coalescence-based Bayesian analysis, using single or multi-locus strategies, reinforced the subdivision of the *M. projectans* complex into at least three clusters, with an exact correspondence to the three lineages previously defined using DNA Barcoding. These results allowed us to reject the hypothesis that the clades observed by Machado et al. [25] would be a result of selective scanning associated with three different and recent invasions of *Wolbachia* in *M. projectans* [28]. Nevertheless, it is still possible that such patterns are resultant of ancient invasions independently fixed in different populations, that were first associated with cytoplasmic incompatibility, and post-zygotic isolation, and later enhanced the emergence or pre-zygotic isolation through reinforcement [91-93]. In this case, *Wolbachia* could act as a pro-speciation factor, and such a longstanding infection could result in fixation also for nuclear alleles. In fact, there is evidence for *Wolbachia* infection in at least one species of the *Zygothrica* group, and this endosymbiont was shown to be more frequent in mycophagous than non-mycophagous Diptera [29]. Although simple diagnostic amplification could be used to confirm the presence of *Wolbachia* in our samples, shortages related to the amplification success caused by some of the markers here did not allow estimation of the precise *Wolbachia* infection rates. Thus, it remains to be further evaluated whether *Wolbachia* was involved in the diversification of the *M. projectans* complex. Even so, this uncertainty does not affect the conclusion about the presence of at least three species under the general morphotype previously ascribed to *M. projectans*.

~~There were some subtle differences among species for abdominal color patterns of~~ *~~M. projectans~~* ~~affinis 2 and the aedeagus type of~~ *~~M. projectans~~* ~~affinis 1. However, no single character allowed distinguishing the three species of the complex. In this case,~~ *~~M. projectans~~* ~~affinis 1 presents abdominal pattern B (Fig 2A, B), and aedeagus type II (Fig 3B);~~ *~~M. projectans~~* ~~affinis 2 presents abdominal pattern A (Fig 2C), and aedeagus type I (Fig 3A); and~~ *~~M. projectans~~* ~~affinis 3 presents abdominal pattern B (Fig 2A, B), and aedeagus type I (Fig 3A). Doubts about the phylogenetic relationships among the three species do not allow to establish the transformation series of these characters within the phylogeny of the~~ *~~M. projectans~~* ~~complex.~~

~~These two characters also allow supporting the identification of~~ *~~M. projectans~~* ~~affinis 2 as the true~~ *~~M. projectans~~*~~. In fact, this species closely resembles the description of~~ *~~M. projectans~~* ~~provided by Sturtevant [94], the redescription of Wheeler and Takada [22] and the holotype photos provided by the National Museum of Natural History (Washington D.C., USA), and matches the aedeagus illustration provided by Wheeler & Takada [22].~~ ~~Furthermore,~~ *~~M. projectans~~* ~~was described for a locality of the Dominican Republic, on the Caribbean Islands, and~~ *~~M. projectans~~* ~~affinis 2 is the species presenting higher suitability for this region [~~~~mean logistic suitability of 0.67, against 0.57 and 0.50 presented for~~ *~~M. projectans~~* ~~affinis 1 and~~ *~~M. projectans~~* ~~affinis 1, respectively (F = 4.74, p < 0.05, in an ANOVA performed with suitability values presented by each species across 25 random points chosen around the area).~~

Another remarkable characteristic of the *M. projectans* complex is the recurrent sympatry and syntopy found among the cryptic species as observed here in three samples from the Atlantic Forest and from the Pampa biome. The coexistence of cryptic species seems to be a common pattern in *Mycodrosophila* since Lacy [95] also reported syntopy for two cryptic species in the Nearctic (*M. claytonae* A and B). Within the *Zygothrica* genus group, the co-occurrence of cryptic species has also been reported for Neotropical species of the genera *Zygothrica* and *Hirtodrosophila* [25, 96]. The presence of preferential mating, in which some males preferentially attract conspecific females to the fruiting bodies of macroscopic fungi, has also been suggested for other species of the *Zygothrica* genus group, and some species of the *Zygothrica* have pronounced degrees of sexual dimorphism [21]. Pronounced levels of prezygotic isolation would also be expected under reinforcement associated with independent invasions of *Wolbachia* in ancestral populations of *M. projectans* (see above).

Besides the frequent sympatry and the general morphological similarity, patterns of biotic and abiotic niches were also quite similar among the three species. Use of the same genera of fungi as breeding sites and the patterns of niche overlap supported the hypothesis of niche conservatism for the *M. projectans* complex [26], which demonstrated that niche conservatism seems to be a common pattern in the *Zygothrica* genus group. Despite differences among species concerning the amplitude of potential distribution, neither pairwise comparison between abiotic niches recovered through ENM strategies rejected the null hypothesis of niche equivalency. This result was already expected once the three species are frequently sympatric and syntopic, although further ecological studies are necessary to clarify how these species are using their resources.

Patterns of morphological similarity and niche equivalency seems to have been maintained despite ancient divergence. In this sense, although there were incongruences among gene trees regarding the topology of the relationships within the *M. projectans* complex, the multi-locus strategy used to reconstruct the species tree under a relaxed molecular clock strategy allowed to date the two cladogenesis events to approximately 17-15 Mya ago. As biogegraphical calibrations can overestimate divergence timings, leading to underestimation of rates [98], whereas fossil calibrations can underestimate divergence timings, leading to overestimated rates [99], we preferred to use intermediate values to accommodate these biases. Interestingly, using these priors, we obtained a mean divergence time of 39.7 Mya [95% HPD Interval 24.5 – 55.4 Mya] which is quite similar to the results obtained by Obbard et al. [98] using a mutation rate establish for four-fold degenerate codons, which added further confidence to our results.

Our estimates indicated the diversification of the *M. projectans* complex occurred in the Neogene period, during the Miocene [100]. This contrasts with the pattern commonly found for Drosophilidae, in which cryptic species are recently diverged lineages [96,101,102], that did not have enough time to accumulate diagnosable features. Thus, this study provides an interesting example of morphological conservation despite ancient divergence. Furthermore, this ancient divergence, in view of the similarity of niches evidenced here for the three lineages, allows us to hypothesize that divergence of the species occurred in allopatry or parapatry, with current records of sympatry and syntopy reflecting secondary contact. This hypothesis of allo- or parapatric distribution in more ancient times was also supported by the EBSP analyses, which showed contrasting patterns of demographic fluctuations for all species. In fact, whereas *M. projectans* affinis 1 did not present significant signals of population expansion, such signals were supported for *M. projectans* affinis 2 and *M. projectans* affinis 3 as occurring at different moments of the Middle Pleistocene [100], at approximately 200 and 400 Kya. Such differences would certainly not be expected if species were sympatric, especially in face of the straightforward evidence of niche equivalence.

Further, these three species seem to have expanded quite recently, as shown by the ENMs projected for different moments of the past. The influence of precipitation to the modeling strategies further supported the interference of the climatic oscillations of the Quaternary in the population dynamics of these species, since this period is characterized by alternating periods of dry and wet conditions in the Southern Hemisphere [104, 105]. These results are also concordant with phylogeographic studies performed with other Brazilian drosophilids, which also showed signals of population expansion dated to the Pleistocene [106-108]. This general pattern suggests that several drosophilid species have been subject to demographic oscillations during the glacial-interglacial cycles of this Epoch [106, 109]. However, although for cactophilic species, expansions are frequently dated to glacial periods [106], the effect of glaciations was the opposite for other species, as recently supported for *D. maculifrons* and *D. ornatifrons* [107, 108]. The pattern found here for the *M. projectans* complex adds three more species to the list of Drosophilidae species suggesting recent population expansion in southern Brazil. Although further sampling is necessary to clarify this scenario, especially in other distribution areas, such results certainly help to disentangle some complexities related to the evolution of the *M. projectans* complex.

Even with this recent diversification, some species of the *M. projectans* complex showed significant levels of population structure between the two southernmost Brazilian biomes, the Pampa and Atlantic Forest. The levels of nucleotide and haplotype or allele diversity seen here for the *M. projectans* complex were also considerable higher than those previously found for several other Drosophilidae species [106, 107, 109, 110-113]. In fact, nucleotide diversity for *M. projectans* affinis 3 reached more than 10,000 times the values found for *Drosophila maculifrons*, a species that also showed signals of population expansion dated to the LGM [107].. Thus, this high diversity could be an intrinsic characteristic of the *M. projectans* complex and possibly of other *Mycodrosophila* species. These patterns suggest the occurrence of high levels of gene flow in a Biome dependent manner for at least some of the species, which may encompass an adaptive response to the restrictions imposed by the ephemerality of resources [21, 95]. In this sense, several cases in which haplotypes from different locations were much more similar in the number of mutational steps than some haplotypes found in the same locality were observed in the haplotype networks of *M. projectans* affinis 2. This suggests possible metapopulation dynamics, suggested for other Neotropical *Drosophila* species due to the cyclic fluctuations in the availability of resources [114]. Even so, at least for *M. projectans* affinis 1 and *M. projectans* affinis 3, the Mantel tests recovered a significant correlation between geographical distances and genetic divergences, suggesting the application of an isolation-by-distance pattern of population differentiation.

**Conclusion**

In conclusion, this study supports the existence of at least three cryptic species within the *M. projectans* complex as initially proposed by Machado et al. [25]. As congruent results were obtained for different markers and techniques, it was possible to exclude numts and recent hitchhiking with endosymbiont parasites. Despite diversification of these lineages dated to around 17-15 Mya (Miocene), only subtle morphological differences were encountered among species and abiotic niches were equivalent among them. Although the three species seem to have expanded to the sampled region after the LGM, they showed high levels of genetic diversity, and, in some cases, some level of population structure between Biomes. Despite inherent difficulties in sampling these species, this study represents a step forward in the understanding of potential spatio-temporal and ecological patterns related to their diversification.

**Acknowledgements**

We are grateful to the authorities responsible for Brazilian Ecological Stations, Reserves or Parks and to the private properties owners that allowed us to perform Drosophilidae searches in their area. All these collections were authorized by the Brazilian Ministério do Meio Ambiente (MMA), in the form of the Sistema de Autorização e Informação em Biodiversidade (SISBIO), and we thank them for their commitment to ethical guidelines and conservation efforts concerning Brazilian scientific activities. We would also like to thank to Dr. Eduardo Bernardi for the fungi identification and to M.Sc. João Pedro Junges dos Santos for his help in drosophilids identification.

**Funding**

This study was supported by Universal-CNPq 14/2013, process numbers 471174/2013-0 and 472973/2013-4. S. M. and M. H. B had a research grant from Coordenação de Aperfeiçoamento de Pessoal de Nível Superior - CAPES (Finance Code 001). L.J.R. is research fellow of CNPq – PQ (# 308371/2018-6).

**References**

1. Lewinsohn TM, Prado PI. How many species are there in Brazil? *Conservation Biology.* 2005; 19:619–624.

2. Giam X, Scheffers BR, Sodhi NS, Wilcove DS, Ceballos G, Ehrlich PR. Reservoirs of richness: least disturbed tropical forests are centres of undescribed species diversity. *Proceedings of the Royal Society of London Series B.* 2012; 279:67–76. DOI: [10.1098/rspb.2013.0879](https://dx.doi.org/10.1098%2Frspb.2013.0879)

3. Morrone JJ. Biogeographis areas and transition zones of Latin America and The Carribean islands based on panbiogeographic and cladistic analyses of the entomofauna. *Annual Review of Entomology.* 2006; 51:467–94. DOI:10.1146/annurev.ento.50.071803.130447

4. Myers N, Mittermeier RA, Mittermeier CG, da Fonseca GAB, Kent J. Biodiversity hotspots for conservation priorities. *Nature.* 2000; 403:853–858. DOI:[10.1038/35002501](https://doi.org/10.1038/35002501)

5. Bennett KD. Continuing the debate on the role of Quaternary environmental change for macroevolution. *Philosophical Transactions of the Royal Society, Series B: Biological Sciences.* 2004; 359:295–303. DOI:[10.1098/rstb.2003.1395](https://doi.org/10.1098/rstb.2003.1395)

6.Rull V. Speciation timing and Neotropical biodiversity: the Tertiary–Quaternary debate in the light of molecular phylogenetic evidence. *Molecular Ecology.* 2008; 17:2722–2729. DOI:10.1111/j.1365-294X.2008.03789.x.

7. Rull V. Neotropical biodiversity: timing and potential drivers. *Trends in Ecology and Evolution.* 2011; 26:508-513. DOI:[10.1016/j.tree.2011.05.011](https://doi.org/10.1016/j.tree.2011.05.011)

8. Freeland JR., Kirk H, Petersen S. Molecular Ecology. Oxford, Wiley-NlackNelll. 2011.

9. Clare EL, Lim BK, Fenton MB, Hebert PDN. Neotropical Bats: estimating species diversity with DNA Barcodes. *PLoS ONE.* 2011; 6:e22648. DOI:[10.1371/journal.pone.0022648](https://doi.org/10.1371/journal.pone.0022648)

10. Crawford A J, Cruz C, Griffth D, Ross H., Ibáñez, R, Lips KR, et al. DNA barcoding applied to *ex situ* tropical amphibian conservation programme reveals cryptic diversity in captive populations. *Molecular Ecology Resources.* 2012; 13(6):1005-18. DOI:10.1111/1755-0998.12054

11. Domingos FMCB, Colli GR, Lemmon A, Lemmon EM, Beheregaray LB. In the shadows: Phylogenomics and coalescent species delimitation unveil cryptic diversity in a Cerrado endemic lizard (Squamata: *Tropidurus*). *Molecular Phylogenetics and Evolution.* 2017; 107:455-465. DOI:[10.1016/j.ympev.2016.12.009](https://doi.org/10.1016/j.ympev.2016.12.009)

12. Janzen D, Burns JM, Qian C, Hallwachsa W, Dapkeya T, Manjunathe R, et al. Nuclear genomes distinguish cryptic species suggested by their DNA barcodes and ecology. *PNAS.* 2017; 114(31):8313-8318. DOI:10.1073/pnas.1621504114

13. Bickford D, Lohman JD, Sodhi NS, Ng PKL, Meier R, Winker K, et al. Cryptic species as a window on diversity and conservation. *Trends in Ecology and Evolution.* 2006; 22:148-155. DOI:[10.1186/jbiol60](https://dx.doi.org/10.1186%2Fjbiol60)

14. Henry CS. Singing and cryptic speciation in insects. *Trends IN Ecology and Evolution.* 1994; 9:388–392.

15. Throckmorton LH. The phylogeny, ecology and geography of *Drosophila*. In: King, R.C. (Ed.), Handbook of Genetics. Plenun, New York. 1975. 421-469.

16. Markow TA, O’Grady P. Reproductive ecology of *Drosophila*. *Functional Ecology.* 2008; 22:747-759. DOI:[10.1111/j.1365-2435.2008.01457.x](https://doi.org/10.1111/j.1365-2435.2008.01457.x)

17. Bächli G. Taxodros database: The database on taxonomy of Drosophilidae. 2021. Available from: http://www.taxodros.unizh.ch/.

18. Carson HL. The ecology of *Drosophila* breeding sites. Harold L. Lyon Arboretum Lecture Number Two. The University of Hawaii, Honolulu. 1971. pp.1–27.

19. Powell JR. Progress and prospects in evolutionary biology: the *Drosophila* model. New York: Oxford University Press. 1997.

20. Grimaldi, DA. Phylogenetics and taxonomy of *Zygothrica* (Diptera: Drosophilidae). *Bulletin of the American Museum of Natural History.* 1987; 186:103–268.

21. Courtney SP, Kibota KK, Singleton TA. Ecology of mushroom-feeding Drosophilidae. *Advances in Ecological Research.* 1990; 20:225–274.

22. Wheeler MR, Takada H. A revision of the American species of Mycodrosophila (Diptera, Drosophilidae). *Annals of the Entomological Society of America.* 1963; 56:392–399.

23. McEvey SF, Polak M. *Mycodrosophila* (Diptera: Drosophilidae) of Fiji and Vanuatu With Description of Nine New Species. *Bishop Museum Occasional Papers.2005;* 84, 35–67.

24. Junges J, Gottschalk MS, Loreto EL, Robe LJ. Two new species of Mycodrosophila (Diptera, Drosophilidae) proposed by molecular and morphological approaches, with a key to American species. *Revista Brasileira de Entomologia.* 2016; 60(1):30-39. DOI:[10.1016/j.rbe.2015.11.008](https://doi.org/10.1016/j.rbe.2015.11.008)

25. Machado S, Dos Santos JP, Fonseca PM, Bolzan AR, David J, Loreto ELS, et al. Neotropical mycophagous drosophilids (Diptera, Drosophilidae): DNA barcoding as a way of overcoming the taxonomic impediment. *Insect Conservation and Diversity.* 2017; 10(03):271-281. DOI:[10.1111/icad.12223](https://doi.org/10.1111/icad.12223)

26. Robe LJ, Machado S, Bolzan AR, dos Santos JPJ, Valer F, dos Santos AP, Blauth ML, et al. Comparative ecological niche modeling and evolutionary ecology of Neotropical mycophagous Drosophilidae (Diptera) species. *Studies on Neotropical Fauna and Environment.* 2014; 49(2):79-94. DOI:[10.1080/01650521.2014.925370](https://doi.org/10.1080/01650521.2014.925370)

27. Richly E, Leister D. NUMTs in Sequenced Eukaryotic Genomes. *Molecular Biology and Evolution.* 2004; 21(6):1081–1084. DOI:[10.1093/molbev/msh110](https://doi.org/10.1093/molbev/msh110)

28. Hurst GDD, Jiggins FM. Problems with mitochondrial DNA as a marker in population, phylogeographic and phylogenetic studies: the effects of inherited symbionts. *Proceedings of the Royal Society B-Biological Sciences.* 2005; 272:1525–1534. DOI:[10.1098/rspb.2005.3056](https://doi.org/10.1098/rspb.2005.3056)

29. Stahlhut J, Clark ME, Baldo L, Russel J, Werren JH, Jaenike J. The mushroom habitat as an ecological arena for global exchange of *Wolbachia*. *Molecular Ecology.* 2010; 19:1940–1952. DOI:[10.1111/j.1365-294x.2010.04572.x](https://doi.org/10.1111/j.1365-294x.2010.04572.x)

30. Linhares S, Gewandsznajder F. Biologia Hoje - Vol 3. São Paulo: ed. Ática. 1998.

31. Câmara IG. Brief history of conservation in the Atlantic Forest. In: GalindoLeal, C., Câmara, I.G. (Eds.), The Atlantic Forest of South America: Biodiversity Status, Threats, and Outlook. CABS and Island Press, Washington. 2003. pp. 31–42.

32. Roesch LFW, Vieira FCB, Pereira VA, Schünemann AL, Teixeira IF, Senna AJT, et al. The Brazilian pampa: a fragile biome. *Diversity*. 2009; 1:182-198

33. Machado S, dos Santos JPJ, Robe LJ, Loreto ELS. An efficient and cheap entomological aspirator to collect mycophylic and anthophilic adult Drosophilidae flies. *Drosophila Information Service.* 2014; 97:169–171.

34. Sassi KA, Heredia F, Loreto ELS, Valente VLS, Rohde C. Transposable elements *P* and *gypsy* in natural populations of *Drosophila willistoni. Genetics and Molecular Biology.* 2005; 28(4):734-739. DOI:10.1590/S1415-47572005000500013

35. Simon C, Frati F, Beckenbach A, Crespi B, Liu H, Flook P. Evolution, weighting, and phylogenetic utility of mitochondrial gene sequences and a compilation of conserved polymerase chain reaction primers. *Annual Review of Entomology.* 1994; 87:651-701. DOI:[10.1093/aesa/87.6.651](https://doi.org/10.1093/aesa/87.6.651)

36. Folmer O, Black M, Hoeh W, Lutz R, Vrijenhoek R. DNA primers for amplification of mitochondrial cytochrome c oxidase subunit I from diverse metazoan invertebrates. *Molecular Marine Biology and Biotechnology.* 1994; 3(5):294-299.

37. Mota NR*,* Robe LJ*,* Valente VLS*,* Budnik M, Loreto ELS. Phylogeny of the *Drosophila mesophragmatica* group (Diptera, Drosophilidae): an example of Andean evolution*. Zoological Science.* 2008; 25:526*–*532*.* DOI:10.2108/zsj.25.526

38. Robe LJ, Loreto EL, Valente VLS. Radiation of the *Drosophila* subgenus (Drosophilidae, Diptera) in the Neotropics. *Journal of Zoological Systematics and Evolutionary Research.* 2010; 48:310–321. DOI:[10.1111/j.1439-0469.2009.00563.x](https://doi.org/10.1111/j.1439-0469.2009.00563.x)

39. Tatarenkov A, Kwiatowski J, Sharecky D, Barrío E, Ayala FJ. On the evolution of Dopa decarboxylase (Ddc) and *Drosophila* systematics*. Jouranl of Molecular Evolution.* 1999; 48:445– 462. DOI:10.1007/PL00006489

40. Staden R. The Staden sequence analysis package. *Molecular Biotechnology.* 1996; 5(3):233-241.

41. Kumar S, Stecher G, Li M, Knyaz C, Tamura K. MEGA X: Molecular Evolutionary Genetics Analysis across Computing Platforms. *Molecular Biology and Evolution.* 2018; 35(6):1547-1549. DOI:[10.1093/molbev/msy096](https://doi.org/10.1093/molbev/msy096)

42. Stephens M, Smith N, Donnelly P. A new statistical method for haplotype reconstruction from population data. American Journal of Human Genetics. 2001; 68:978-989.

43. Stephens M, Donnelly P. A comparison of Bayesian methods for haplotype reconstruction from population genotype data. American Journal of Human Genetics. 2003; 73:1162-1169.

44. Rozas J, Ferrer-Mata A, Sánchez-DelBarrio JC, Guirao-Rico S, Librado, P, Ramos-Onsins SE, et al. DnaSP 6: DNA Sequence Polymorphism Analysis of Large Datasets. *Molecular Biology and Evolution.* 2017; 34:3299-3302. DOI:10.1093/molbev/msx248

45. Kimura M. A simple method for estimating evolutionary rate of base substitutions through comparative studies of nucleotide sequences. *Journal of Molecular Evolution.* 1980; 16:111-120.

46. Robe LJ, Machado S, Bartholomei-Santos ML. The DNA Barcoding and the caveats with respect to its application to some species of Palaemonidae (Crustacea, Decapoda). Zoological Science. 2012; 29:714–724. DOI:[10.2108/zsj.29.714](https://doi.org/10.2108/zsj.29.714)

47. Klaus AV, Kulasekera VL, Schawaroch V. Three-dimensional visualization of insect morphology using confocal laser scanning microscopy. *Journal of Microscopy.* 2003; 212:107-121. DOI: [10.1046/j.1365-2818.2003.01235.x](https://doi.org/10.1046/j.1365-2818.2003.01235.x)

48. Burla H. Die Drosophidengattung *Zygothrica* und ihre beziehung zur drosophilauntergattung *Hirtodrosophila*. Mitt Zool Mus Berl. 1956; *32:*90–321

49. Frota-Pessoa O. Sobre o subgênero *Hirtodrosophila*, com descrição de uma nova espécie (Dipt., Drosophilidae, *Drosophila*). Rev Bras Entomol. 1945; 5:469-483

50. Grimaldi DA. *Hirtodrosophila* of North America (Diptera: Drosophilidae). Bull Am Mus Nat Hist. 2018; 421:1-75.

51. Vilela CR, Bachli G. Taxonomic studies on Neotropical species of seven genera of Drosophilidae (Diptera). Mitt. Schweiz. entomol. Ges. 1990; 63:1-332

52. Vilela CR, Bachli G. Revision of the Neotropical genus *Paraliodrosophila* (Diptera, Drosophilidae). Mitt Schweiz Entomol Ges. 2007; 80:291–317. DOI:10.5169/seals-402953

53. Ratnasingham S, Hebert PDN. BOLD: The Barcode of Life Data System ([http://www.barcodinglife.org](http://www.barcodinglife.org/)). Molecular Ecology Notes. 2007; 7(3):355–364. DOI:[10.1111/j.1471-8286.2007.01678.x](https://dx.doi.org/10.1111%2Fj.1471-8286.2007.01678.x)

54. Akaike H. A new look at the statistical model identification. *IEEE Transactions on Automatic* Control. 1974; 19:716–723.

55. Lanfear R, Frandsen PB, Wright AM, Senfeld T, Calcott B. PartitionFinder 2: New Methods for Selecting Partitioned Models of Evolution for Molecular and Morphological Phylogenetic Analyses. Molecular Biology and Evolution. 2016; 34(3):772–773. DOI:[10.1093/molbev/msw260](https://doi.org/10.1093/molbev/msw260)

56. Ronquist F, Teslenko M, van der Mark P, Ayres DL, Darling A, Höhn S, et al. MrBayes 3.2: Efficient Bayesian Phylogenetic Inference and Model Choice Across a Large Model Space. *Systematic Biology.* 2012; 61(3):539–542. DOI:[10.1093/sysbio/sys029](https://dx.doi.org/10.1093%2Fsysbio%2Fsys029)

57.Pons J, Barraclough T, Gomez-Zurita J, Cardoso A, Duran D, Hazell S, et al. Sequence-Based Species Delimitation for the DNA Taxonomy of Undescribed Insects. Systematic Biology. 2006; 55:595–609. DOI:10.1080/10635150600852011

58. Fujisawa T, Barraclough TG. Delimiting species using single-locus data and the generalized mixed yule coalescent approach: a revised method and evaluation on simulated data sets. *Systematic Biology.* 2013; 62:707–24. DOI:10.1093/sysbio/syt033

59. Jones G, Aydin Z, Oxelman B. DISSECT: an assignment-free Bayesian discovery method for species delimitation under the multispecies coalescent. *Bioinformatics.* 2014; 31(7):991–998. DOI:10.1093/bioinformatics/btu770

60. Guindon S, Gascuel O. A simple, fast and accurate method to estimate large phylogenies by maximum-likelihood. *Systematic Biology.* 2003; 52:696-704. DOI:[10.1080/10635150390235520](https://doi.org/10.1080/10635150390235520)

61. Darriba D, Taboada GL, Doallo R, Posada D. jModelTest 2: more models, new heuristics and parallel computing. *Nature Methods.* 2012; 9(8):772. DOI:10.1038/nmeth.2109

62. Bouckaert R, Vaughan TG, Barido-Sottani J, Duchêne S, Fourment M, Gavryushkina A, et al. BEAST 2.5: An advanced software platform for Bayesian evolutionary analysis. *PloS* [*C*](https://doi.org/10.1371/journal.pcbi.1006650)*omputational* [*B*](https://doi.org/10.1371/journal.pcbi.1006650)*iology.* 2019; 15(4):e1006650. DOI:10.1371/journal.pcbi.1006650

63. R Core Team. R: A language and environment for statistical computing. R Foundation for Statistical Computing, Vienna, Austria. 2017. Available from [https://www.R-project.org/](https://www.r-project.org/).

64. Bouckaert R, Heled J, Kühnert D, Vaughan T, Wu C-H, Xie D, et al. BEAST 2: A Software Platform for Bayesian Evolutionary Analysis. *PLoS Computational Biology.* 2014; 10(4):e1003537. DOI:10.1371/journal.pcbi.1003537

65. Rambaut A, Suchard MA, Xie D, Drummond AJ. Tracer v1.6. 2014. Available from <http://beast.bio.ed.ac.uk/Tracer>

66. Jones G. Algorithmic improvements to species delimitation and phylogeny estimation under the multispecies coalescent. *Journal of Mathematical Biology.* 2017; 74:447-467. DOI:10.1007/s00285-016-1034-0

67. Puillandre N, Lambert A, Bouillet S, Achaz G. ABGD, Automatic Barcode Gap Discovery for primary species delimitation. Molecular Ecology. 2012; 21(8):1864-1877. DOI:[10.1111/j.1365-294X.2011.05239.x](https://doi.org/10.1111/j.1365-294X.2011.05239.x)

68. Brown SDJ, Collins RA, Boyer S, Lefort M-C, Malumbres-Olarte J, Vink CJ, et al. SPIDER: an R package for the analysis of species identity and evolution, with particular reference to DNA barcoding. *Molecular Ecology Resources.* 2012; 12:562-565. DOI:[10.1111/j.1755-0998.2011.03108.x](https://doi.org/10.1111/j.1755-0998.2011.03108.x)

69. Rohlf FJ. tpsDig2, digitize landmarks and outlines, version 2.10. Dept Ecology & Evolution, St. Univ. New York, Stony Brook, New York. 2006.

70. Rohlf FJ. tpsRelw, relative warps analysis, version5/13/05. Dept Ecology & Evolution, St. Univ. New York, Stony Brook, New York. 2007.

71. Hammer Ø, Harper DAT, Ryan PD. PAST: Paleontological Statistics Software Package for Education and Data Analysis. *Palaeontologia Electronica.* 2001; 4(1):9.

72. Tajima F. Statistical method for testing the neutral mutation hypothesis by DNA polymorphism. *Genetics.* 1989; 123:585–595.

73. Fu YX*.* Statistical tests of neutrality of mutations against population growth, *hitchhiking* and background selection*. Genetics.* 1997; 147:915*–*925*.*

74. Librado P, Rozas J. DnaSP v5: a software for comprehensive analysis of DNA polymorphism data*. Bioinformatics.* 2009; 25:1451*–*1452*. DOI:10.1093/bioinformatics/btp187*

75. Bandelt H-J, Forster P, Rohl A. Median-joining networks for inferring intraspecific phylogenies. *Molecular Biology and Evolution.* 1999; 16: 37-48. DOI:[10.1093/oxfordjournals.molbev.a026036](https://doi.org/10.1093/oxfordjournals.molbev.a026036)

76. Excoffier L, Licher HEL. Arlequin suite ver 3.5: A new series of programs to perform population genetics analyses under Linux and Windows. *Molecular Ecology Resources.* 2010; 10:564-567. DOI:10.1111/j.1755-0998.2010.02847.x.

77. Corander J, Marttinen P, Mäntyniemi S. Bayesian identification of stock mixtures from molecular marker data. Fishery Bulletin. 2006; 104:550-558.

78. Yassin A. Phylogenetic classification of the Drosophilidae rondani (Diptera): the role of morphology in the postgenomic era. Systematic Entomology. 2013; 38:349–364. DOI: 10.1111/j.1365-3113.2012.00665.x

79. Grimaldi DA. Relicts in the Drosophilidae (Diptera). In Liebherr, J. K.(ed.), Zoogeography of Caribbean insects. Ithaca: Cornall Univ. Press. 1988. pp.183-213.

80. Iturralde-Vinent MA, MacPhee RDE. Age and paleogeographical origin of Dominican amber. Science. 1996; 273:1250-1252.

81. Tamura K, Subramanian S, Kumar S. Temporal patterns of fruit fly (*Drosophila*) evolution revealed by mutation clocks. *Molecular Biology and Evolution.* 2004; 21, 36–44. DOI:[10.1093/molbev/msg236](https://doi.org/10.1093/molbev/msg236)

82. Suvorov A, Kim BY, Wang J, Armstrong EE, Peede D, D` Agostino ERR, et al. Widespread introgression across a phylogeny of 155 Drosophila genomes. BioRxiv, preprint. 2021. DOI: 10.1101/2020.12.14.422758

83. Drummond AJ, Rambaut A. BEAST: Bayesian evolutionary analysis by sampling trees. *BMC Evolutionary Biology.* 2007; 7:214.

84. Rambaut A. FigTree v1.3.1. Institute of Evolutionary Biology, University of Edinburgh, Edinburgh. 2010. Available from http://tree.bio.ed.ac.uk/software/figtree/

85. Elith J, Leathwick JR. Species distribution models: ecological explanation and prediction across space and time. *Annual Review Of Ecology, Evolution, and Systematics.* 2009; 40:677-697. DOI:[10.1146/annurev.ecolsys.110308.120159](https://doi.org/10.1146/annurev.ecolsys.110308.120159)

86. Brown JL, Hill DJ, Dolan AM, Carnaval AC, Haywood AM. PaleoClim, high spatial resolution paleoclimate surfaces for global land areas. *Scientific Data.* 2018; 5: 180254. DOI:[10.1038/sdata.2018.254](https://dx.doi.org/10.1038%2Fsdata.2018.254)

87. Hijmans RJ, van Etten J. Raster: Geographic analysis and modeling with raster data. R package version 2.0-12. 2012. Available from http://CRAN.R-project.org/package=raster

88. Wei T, Simko V. R package "corrplot": Visualization of a Correlation Matrix (Version 0.84).2017. Available from <https://github.com/taiyun/corrplot>

89. Hijmans RJ, Phillips S, Leathwick J, Elith J. Dismo: Species Distribution Modeling. R Package Version 1.3-3. 2020.

90. Warren DL, Matzke N, Cardillo M, Baumgartner J, Beaumont L, Huron N, et al. ENMTools (Software Package). 2019. DOI:10.5281/zenodo.3268814

91. Werren JH. Wolbachia and speciation. Endless forms: species and speciation. 1998; 245-260.

92. Bordenstein SR. 17 Symbiosis and the Origin of Species. Insect symbiosis. *2003;* 283.

93. Telschow A, Hammerstein P, Werren JH. The effect of Wolbachia versus genetic incompatibilities on reinforcement and speciation. Evolution. 2005; *59*(8), 1607-1619.

94. Sturtevant AH. Notes on North American Drosophilidae with descriptions of twenty-three new species. Annals of the Entomological Society of America. 1916; 9:323-343.

95. Lacy RC. Niche breadth and abundance as determinants of genetic variation in populations of mycophagous drosophilid flies (Diptera: Drosophilidae). *Evolution.* 1982; 36(6):1265-1275.

96. Fonseca PM, Loreto EL, Gottschalk MS, Robe LJ. Cryptic diversity and speciation in the Zygothrica genus group (Diptera, Drosophilidae): the case of Z. vittimaculosa Wiedemann. *Insect Systematics & Evolution. 2017;* 48(3):285-313. DOI:[10.1163/1876312X-48022158](https://doi.org/10.1163/1876312X-48022158)

97. Carson HL, Clague DA. Geology and biogeography of the Hawaiian Islands. In: Wagner, W., Funk, V. (Eds.), Hawaiian Biogeography: Evolution in a Hotspot Archipelago. 1995. Smithsonian Institution Press, Washington, DC, pp. 14–29.

98. Obbard DJ, Maclennan J, Kim K-W, Rambaut A, O`Grady PM, Jiggins FM. Estimating Divergence Dates and Substitution Rates in the Drosophila Phylogeny. Molecular Biology and Evolution. 2012; 29(11): 3459–3473. DOI: 10.1093/molbev/mss150

99. Yang Z, Rannala B. Bayesian estimation of species divergence times under a molecular clock using multiple fossil calibrations with soft bounds. 2006. *Molecular biology and evolution*, *23*(1), 212-226.

100. Cohen KM, Finney SC, Gibbard PL, Fan J-X. The ICS International Chronostratigraphic Chart. *Episodes.* 2013; 36:199-204. DOI:10.18814/epiiugs/2013/v36i3/002

101. Robe LJ, Cordeiro J, Loreto ELS, Valente VLS. Taxonomic boundaries, phylogenetic relationships and biogeography of the *Drosophila willistoni* subgroup (Diptera: Drosophilidae). *Genetica*. 2010; 138(6):601-617. DOI:10.1007/s10709-009-9432-5

102. Castro Vargas C, Richmond MP, Ramirez Loustalot Laclette M, Markow TA Early events in speciation: cryptic species of *Drosophila aldrichi*. *Ecology and Evolution*. 2017; 7(12):4220-4228. DOI: 10.1002/ece3.2843

103. Bickford D, Lohman DJ, Sodhi NS, Ng PKL, Meier R, Winker K, Ingram KK, Das I. Cryptic species as a window on diversity and conservation. *Trends in Ecology & Evolution.* 2007; 22(3):148-155. DOI: 10.1016/j.tree.2006.11.004.

104. Haffer J. Speciation in Amazonian forest birds. *Science.* 1969; 165:131-137

105. Ab'Saber AN. Os domínios morfoclimáticos na América do Sul. *Geomorfologia.* 1977; 52:1-22.

106. Franco FF, Manfrin MH*.* Recent demographic history of cactophilic *Drosophila* species can be related to Quaternary palaeoclimatic changes in South America*. Journal of Biogeography.* 2013; 40:142*–*154*.* DOI:[10.1111/j.1365-2699.2012.02777.x](https://doi.org/10.1111/j.1365-2699.2012.02777.x)

107. De Ré FC, Gustani EC, Oliveira APF, Machado LPB, Mateus RP, Loreto ELS, et al. Brazilian populations of *Drosophila maculifrons* (Diptera: Drosophilidae): low diversity levels and signals of a population expansion after the Last Glacial Maximum. *Biological Journal of the Linnean Society.* 2014; 112:55–66. DOI:[10.1111/bij.12244](https://doi.org/10.1111/bij.12244)

108. Gustani E, Oliveira AP, Santos MS, Machado LB, Rogério PM. Demographic Structure and Evolutionary History of Drosophila ornatifrons (Diptera, Drosophilidae) from Atlantic Forest of Southern Brazil. *Zoological Science.* 2015; 32(2):141-150. DOI:[10.2108/zs140062](https://doi.org/10.2108/zs140062)

109. Moraes EM, Yotoko KSC, Manfrin MH, Solferini VN, Sene FM. Phylogeography of the cactophilic species *Drosophila gouveai*: demographic events and divergence timing in dry vegetation enclaves in eastern Brazil*. Journal of Biogeography.* 2009; 36:2136–2147. DOI:[10.1111/j.1365-2699.2009.02145.x](https://doi.org/10.1111/j.1365-2699.2009.02145.x)

110. De Brito RA., Manfrin MH, Sene FM. Mitochondrial DNA phylogeography of Brazilian populations of Drosophila buzzatii. *Genetics and Molecular Biology.* 2012; 25:161–171. DOI:10.1590/S1415-47572002000200009

111. Hurtado LA*,* Erez T*,* Castrezana S, Markow TA*.* Contrasting population genetic patterns and evolutionary histories among sympatric Sonoran Desert cactophilic *Drosophila. Molecular Ecology.* 2014; 13*:*1365*–*1375. DOI:[10.1111/j.1365-294X.2004.02169.x](https://doi.org/10.1111/j.1365-294X.2004.02169.x)

112. Reed LK, Nyboer M, Markow TA. Evolutionary relationships of Drosophila mojavensis geographic host races and their sister species Drosophila arizonae. *Molecular Ecology.* 2007; 16:1007–1022. DOI:[10.1111/j.1365-294X.2006.02941.x](https://doi.org/10.1111/j.1365-294X.2006.02941.x)

113. Mirol PM*,* Routtu J*,* Hoikkala A, Butlin RK*.* Signals of demographic expansion in *Drosophila virilis. BMC Evolutionary Biology*. 2008; 8:59. DOI: 10.1186/1471-2148-8-59

114. De Ré FC, Wallau GL, Robe LJ, Loreto ELS. Characterization of the complete mitochondrial genome of flower-breeding *Drosophila incompta* (Diptera, Drosophilidae). *Genetica*. 2014; 142(6):525-535. DOI: 10.1007/s10709-014-9799-9

115. Löwenberg-Neto P. Neotropical region: a shapefile of Morrone’s (2014) biogeographical

regionalisation. Zootaxa . 2014; 3802(2): 300-300.

116. IBGE. Biomas e Sistemas Marinho-Costeiro do Brasil – 1:250 000. 2019. Available at <https://www.ibge.gov.br/geociencias/cartas-e-mapas/informacoes-ambientais/15842-biomas.html?=&t=publicacoes>.

**Supporting information**

**S1 Fig.** Illustration of the right wing of a specimen of the *M. projectans* complex showing the landmarks chosen for morphometry analysis.

**S2 Fig.** Majority rule consensus tree recovered through Bayesian analysis based on (A) COI, (B) COII, (C) HB and (D) AMD datasets. Branch lengths are proportional to the scale, given in substitutions per site. Values near the internal branches represent the posterior probability of each clade.

**S3 Fig.** Cartesian plot from wings relative warps reported for the different species of the *M. projectans* complex.

**S4 Fig.** Median-joining network reconstructed with COI+COII haplotypes characterized for each species of the *M. projectans* complex: (A) *M. projectans* affinis 1, (B) *M. projectans* affinis 2, and (C) *M. projectans* affinis 3. Each circle represents a different haplotype, whose size is proportional to frequency. Each color represents different sampling points, in accordance with the legend presented on Figure 1. Black small circles represent median vectors. Dashes in the lines connecting different haplotypes represent the number of mutations between them.

**S5 Fig.** Median-joining network reconstructed with (A) HB and (B) AMD phased alleles characterized for each species of the *M. projectans* complex. Each circle represents a different haplotype, whose size is proportional to frequency. Each color represent different sampling points, in accordance with the legend presented on Figure 1. Black small circles represent median vectors. Dashes in the lines connecting different haplotypes represent the number of mutations between them.

**S6 Fig.** Results from the spatial clustering analysis performed by BAPS for each of the three species of the *M. projectans* complex based on (A) COI and (B) COII datasets. Axis x and y reflect the longitude and latitude of each population, whereas each color represent a different cluster of populations.

**S7 Fig.** Extended Bayesian Skyline Plots depicting oscillations of population sizes faced by *M. projectans* affinis 2 (A) and *M. projectans* affinis 3 (B) during the last 1 Mya.

**S1 Table.** List of sampling sites of the *Mycodrosophila projectans* complex, with their respective geographic coordinates.

**S2 Table.** List of specimens used in this study, with their respective origin and GenBank accession numbers.

**S3 Table.** Description of wing landmarks used in morphometric analysis.

**S4 Table.** Results of pairwise comparisons involving Dn and Ds, and the codon-based-Z test of purifying selection performed to assist in the detection of numts for the two mitochondrial datasets.

**S5 Table.** Number of clusters recovered for the *M. projectans* complex by ABGD, GMYC and Stacey under different datasets.

**S6 Table.** List of diagnostic characters recovered for each species of the *M. projectans* complex under the COI and COII datasets. Sites that did not reveal diagnostic nucleotides were omitted.

**S7 Table.** Patterns of morphology found in different specimens of the *M. projectans* complex based on the evaluation of abdominal spots and aedeagus type.

**S8 Table.** Results of ANOVA and CVA of wing variation among the three species of the *M. projectans* complex.

**S9 Table.** Pairwise values of Fst among the three species of the *M. projectans* complex, as calculated for different datasets.

**S10 Table.** Results of the Analyses of Molecular Variance (AMOVA) considering the subdivision of the *M. projectans* complex in three species, as recovered with the use of different datasets.

**S11 Table.** Results of the Mantel tests performed for each of the three species of the *M. projectans* complex with the use of different datasets.

**S12 Table.** List of resources in which each species of the *M. projectans* complex was collected. Sampling point number refers to codes presented on S1 Table.

**S13 Table.** Number of points and mean AUC values presented by the ENM reconstructed for each species of the M. *projectans* complex.

**S14 Table.** Estimates of relative contributions of each of the employed environmental variables to the ENMs reconstructed for each species of the *M. projectans* complex.

**S15 Table.** Pairwise values of abiotic niche overlap estimated among the three species of the *M. projectans* complex according to Schoener’s D (D) (above diagonal) and Hellinger’s (I) (below diagonal) indexes.

**Table Legend:**

**Table 1.** Summary information and essential statistics calculated for each of the three species of the *M. projectans* complex. Sampling points were numbered according to S1 Table.

**Figure Legends:**

**Fig. 1.** Map of sampling points (A) and geographical distribution of COI haplotypes recovered for each species of the *M. projectans* complex: (B) *M. projectans* affinis 1, (C) *M. projectans* affinis 2, and (D) *M. projectans* affinis 3. Brazilian biomes where collections were performed are highlighted on the map, in which squares indicate sampling points where all three species of the *M. projectans* complex were collected in sympatry; triangles indicate sampling points where two species were sampled; and circles represent single species records. Colors represent each different sampling point, according to the legend presented on the Figure. On the Networks, each circle represents a different haplotype, whose size is proportional to frequency, and whose colors refer to each locality on the map. Black small circles represent median vectors. Dashes in the lines connecting different haplotypes represent the number of mutations between them. Neotropical and Brazilian Biomes shapefiles were obtained from Löwenberg-Neto [115] and from IBGE [116], respectively.

**Fig. 2.** Abdominal coloration (dorsal view) of the different species of the *M. projectans* complex, as visualized in a stereomicroscope: (A) Pattern A1, presenting two individual light spots in tergite IV; (B) Pattern A2, presenting two confluent light spots, that are narrowed in the median region, (C) Pattern B, presenting confluent light spots, without the narrowing in the median region. Patterns A1 and A2 are seen in *M. projectans* affinis 1 and *M. projectans* affinis 3, whereas pattern B is exclusive of *M. projectans* affinis 2. Image (A) of sample 577A109, (B) of sample 581A26 and (C) of sample 577A88 (see Table S7 for more details).

**Fig. 3.** Images of the aedeagus structures reported for the different species of the *M. projectans* complex as visualized by confocal miscroscopy: (A) Type I, encountered in *M. projectans* affinis 2 and *M. projectans* affinis 3; (B) Type II, detected for *M. projectans* affinis 1. From top to bottom: lateral, dorsolateral and ventral views. Images (A) of sample 584A35 and (B) of sample 584A39 (see Table S7 for more details).

**Fig. 4.** Phylogenetic trees recovered by Starbeast: (A) COI gene tree, (B) COII gene tree, (C) HB gene tree, (D) AMD gene tree, and (E) multi-locus species tree. Numbers above internal nodes in the gene trees and below the internal nodes in the species tree represent support values, as represented by the posterior probability (PP) of each clade. Numbers above internal nodes in the species tree represent mean divergence dates. Bars within internal nodes represent the confidence intervals (CI).

**Fig. 5.** Environmental distribution models predicted by maximum entropy for each of the three species of the *M. projectans* complex for the (A) Pliocene, (B) Pleistocene (787 kya), (C) Last Interglacial, (D) Last Glacial Maximum, (E) Present. Neotropical shapefile was obtained from Löwenberg-Neto [115], as available at http://purl.org/biochartis/neo2014shp.
