# Supplementary material for: Unveiling the Mycodrosophila projectans (Diptera, Drosophilidae) species complex: Insights into the evolution of three Neotropical cryptic and syntopic species
Source: PLoS One. 2022 May 25;17(5):e0268657. doi: 10.1371/journal.pone.0268657 (PMC9132268; doi:10.1371/journal.pone.0268657)
Supplement: Attachment — Submitted filename: Response to Reviewers.docx [file pone.0268657.s026.docx]

Santa Maria, April 21^th^, 2022.

Dear Dr. William J. Etges,

Please find attached the MS entitled **“Unveiling the Mycodrosophila projectans (Diptera, Drosophilidae) species complex: insights into the evolution of three Neotropical cryptic and syntopic species”** which contains a revised version of the previous submission to PLOS ONE (PONE-D-21-28403). We thank you for the opportunity as well as for the constructive criticism, which allowed several improvements in our study.

We carefully followed the recommendations and provide below the explanation (in bold) on how we proceeded about each specific comment (in italics). Excerpts from the text are represented in yellow in this letter and are highlighted in blue in the tracked version of the MS. Words or sentences that have been pointed to present grammar problems were edited according to suggestions provided in the pdf file and are highlighted in blue in the tracked version of the MS. When there is an overlap of both circumstances, the modified words or sentences are presented in green in the MS. Finally, specific suggestions that could not be accepted are presented in red, and justification for this is provided bellow.

We also detach that the manuscript was proofread by a specialized company (see certificate attached to the submission). Although the format style hired from this company was British, suggestions which did not follow USA English were not implemented.

We are confident the current version is much improved and hope it reaches the level of quality expected by PLOS ONE.

Sincerely,

L. J. Robe, on behalf of all authors

Editor`s comment:

*“The authors have done a good job in responding to the peer reviewers' comments, but the authors did not follow the instructions listed in the initial decision letter to address all of the English language problems. I think the paper contains some interesting and verifiable results, but the Discussion needs to be shortened and all speculation and redescriptions of the Results section need to be eliminated. I have uploaded an edited copy of your MS with many specific changes and questions. Several sections and paragraphs in the Discussion section should be deleted. Overall, the writing style is wordy with much jargon. It reads like a thesis. Again, I suggest the MS be proofread by an English speaker before resubmission because I may not have found all of the grammar and wording problems.”*

**Thank you for your effort in thoroughly reviewing our manuscript. Most of the changes pointed out in the pdf were performed, and the MS was proofread by a specialized company (see certificate attached to the submission). We have also withdrawn several speculations of the Discussion, as well as the redescriptions of the results. Thus, the last section was significantly shortened.**

**Concerning each commentary presented in the pdf:**

Abstract – line 39: *Look up the definition of niche.*

**As suggested, we withdrawn the reference to “biotic and abiotic niches”, so that the mentioned sequence now looks like: “Ecologically, sympatry and syntopy seem to be recurrent for these three cryptic species, which present widely overlapping niches, implying niche conservatism.” (lines 34-36)**

Introduction – lines 69-71:

*“This last sentence needs rewriting or deletion. There are many reasons beyond non-visual signals why cryptic species exist. Why is vision so important? All kinds of signaling systems are known. They could have diverged in allopatry and then converged later.”*

**We decided to rewrite the sentence, as follows: “The mechanisms responsible for this pattern are not well understood [13], but it has been suggested that sexual selection acting at the level of non-visual signals [14], recent divergence [15], chance fixation of alleles related to epistatic incompatibilities [16], and allopatric speciation associated with morphological stasis or convergent evolution [17] may help to explain the ubiquity of this phenomenon.” (lines 56-60)**

Materials and Methods – line 139: *State what you mean by “active period”.*

**The sentence was rewritten to: “In these localities, active searches for fruiting bodies of macroscopic fungi were performed in forest fragments during daylight.” (lines 116-118)**

Results – line 357: *What is “Bold system”? Define*.

**BOLD is defined in line 164 of the Materials and Methods: “All individuals were identified by the DNA barcoding in BOLD (Barcode of Life Data) System [51] using COI sequences.”**

Results – title presented in lines 433-433: replace “What can we say about the differential patterns of genetic diversity and abiotic niches that distinguish the three species and what this suggests about their speciation?” to “Patterns of genetic diversity and abiotic niches distinguish the three species and suggest modes of speciation”.

**The title was rewritten to: “Patterns of distribution of genetic diversity and abiotic niches help to disentangle evolutionary history and suggest modes of speciation” (lines 376-378)**

Discussion – lines 569-591: *These are a restatement of the results. Delete.*

**The paragraph was withdrawn, and only two sentences were left to mention the cryptic morphology: “The cryptic morphology among the three species was confirmed after rigorous analysis, and only a few subtle morphological differences were found for the three species in terms of aedeagus morphology and abdominal color patterns. However, none of these allowed the three species of the complex to be unambiguously distinguished” (lines 499-502). Otherwise, this aspect would not be mentioned in the discussion, and there would be a gap to the comprehension of the next paragraphs.**

Discussion – lines 595-596, 600-601: *Previous sentence deleted. It’s irrelevant and speculative.*

**Both sentences were deleted, as well as all the section dealing with preferential mating. The new section now looks like: “In addition to this cryptic morphology, the M. projectans complex also detaches for recurrent sympatry and syntopy among its species, as observed here in three samples from the Atlantic Forest and the Pampa biome. The coexistence of cryptic species seems to be a common pattern in Mycodrosophila since Lacy [92] also reported syntopy for two cryptic species in the Nearctic (M. claytonae A and B). Within the Zygothrica genus group, the co-occurrence of cryptic species has also been reported for Neotropical species of the genera Zygothrica and Hirtodrosophila [28, 93]” (lines 502-508). This paragraph was further associated with the next one, continuing with: “Nevertheless, for the M. projectans complex, beyond co-occurrence, patterns of biotic and abiotic niches were also quite similar, and species used the same genera of fungi as breeding sites. Despite differences among species concerning the amplitude of potential distribution, neither pairwise comparison between abiotic niches recovered through ENM strategies rejected the null hypothesis of niche equivalency. These results support the hypothesis of niche conservatism for the M. projectans complex [29], suggesting that it may be a common pattern in the Zygothrica genus group. Nevertheless, further ecological studies are necessary to clarify in which way these species use their resources and how competition affects their co-occurrence” (lines 509-516).**

**Lines 624-61, and 632-637 were also deleted, and the two next paragraphs were shortened and associated: “Patterns of morphological similarity and niche equivalency seem to have been maintained despite ancient divergence. In this sense, although there were incongruences among gene trees regarding the topology of the relationships within the M. projectans complex, the multi-locus strategy used to reconstruct the species tree under a relaxed molecular clock strategy allowed us to date the two cladogenesis events to approximately 17–15 Mya ago. These estimates suggest that the diversification of the M. projectans complex occurred in the Neogene period during the Miocene [94]. This contrasts with the pattern commonly found for Drosophilidae, in which cryptic species encompass recently diverged lineages [93, 95, 96] that did not have enough time to accumulate diagnosable features. Thus, this study provides an interesting example of morphological conservation, despite ancient divergence. Furthermore, this ancient divergence, in view of the similarity of niches evidenced here for the three lineages, allows us to hypothesize that divergence of the species occurred in allopatry or parapatry, with current records of sympatry and syntopy reflecting secondary contact. This hypothesis of allo- or parapatric distribution in more ancient times was also supported by EBSP analyses, which showed contrasting patterns of demographic fluctuations for all species. In fact, whereas M. projectans affinis 1 did not present significant signals of population expansion, such signals were supported for M. projectans affinis 2 and M. projectans affinis 3 as occurring at different moments of the Middle Pleistocene [94], ca. 200 and 400 Kya, respectively. Such differences would certainly not be expected if species were sympatric, especially in the face of straightforward evidence of niche equivalence” (lines 517-536)**

Discussion, line 655-656: *Replace “these three species seem to have expanded to the sampled region quite recently” to “these three species seem to have expanded quite recently”.*

**In this sentence, we preferred to adopt an intermediate version stating that “these three species seem to have expanded to Southern Brazil quite recently” (lines 537-538), since our modelling results clearly suggest an expansion to this specific area.**

**Concerning the English grammar corrections suggested on the pdf and the spelling review, most suggestions were accepted, and are detached in yellow or green in the text with tracked changes. There were only some specific cases in which the suggestion was not followed, and these cases are marked in red in the text with tracked changes. Bellow we detail each particular situation (see that lines reference refer to the reviewed version):**

Abstract:

→ Line 25: *Replace “The Zygothrica genus group” by “The Zygothrica species group” 🡪* **This replacement would not be correct, since the target taxon is really a genus group, as proposed by Grimaldi (1990).**

→ Line 25: “*to be a speciose group” by “to be speciose”* 🡪 **Ok.**

→ Line 26: “*have revealed” by “have been”* 🡪 **Ok.**

→ Line 28: “*The aim of this study is…” by “The aim of this study was”* 🡪 **Ok.**

→ Line 32: “… *described for…” by “described as”* 🡪 **Ok.**

→ Lines 33-34: *“**...In fact, only some subtle morphological differences could be encountered for the three species when aedeagus patterns are evaluated in association with abdominal color patterns.”* **was replaced by “Only a few subtle morphological differences were found for the three species in terms of aedeagus morphology and abdominal color patterns”, as requested.**

→ Lines 33-34: “… seems to be a recurrent pattern…” ***was replaced by “seem to be recurrent”*, as suggested.**

→ Lines 35-36: “… *which present widely overlapped biotic and abiotic niches, in an intrinsic pattern of niche conservatism.”* **was replaced by “which present widely overlapping niches, implying niche conservatism.”**

→ Lines 36-37: *Replace* “*This straightforward morphological and ecological similarity occurs even though cladogenesis” by “This morphological and ecological similarity has persisted though cladogenesis”* 🡪 **Ok.**

→ Lines 37*: Replace “seems to have taken place during Miocene” by “dates back to the Miocene”* 🡪 **Ok.**

→ Line 39*: Replace “added to some contrasting patterns” by “in addition to contrasting patterns”* 🡪 **Ok.**

→ Lines 39-40*: Replace “allowed hypothesizing allopatric or parapatric diversification” to “allowed us to hypothesize patterns of allopatric or parapatric diversification”* 🡪 **Ok.**

→ Lines 40-41*: Replace “with expansion to the sampled region leading to secondary contact” by “with expansion within their current geographic ranges” 🡪* **Because our samples did not encompass the whole geographic ranges of each species, we preferred to change this sentence to “with secondary contact in Southern Brazil”.**

Introduction:

→ Line 49: *“...the world biodiversity…”* **was changed to “...the world’s biodiversity...”, as suggested.**

→ Line 51: “.*.. processes responsible for the origin of all this diversity have been largely discussed…*” **was changed to … “processes responsible for the origin of this diversity have been previously discussed...”, as suggested.**

→ Line 52-53: *“The molecular taxonomy…”* **was changed to “Molecular taxonomy…”, as requested.**

→ Line 57: *Replace “largely misunderstood” by “not well understood”* **🡪 Ok.**

→ Line 57: *Replace “Nevertheless” by “but”* **🡪 Ok.**

→ Line 61: *Replace* *“Drosophilidae is a speciose family of insects, presenting …” by “The family Drosophilidae includes…”* **🡪 Ok.**

→ Line 62: *Replace* *“Flies of this family are …” by “Different species are…”* **🡪 Ok.**

→ Line 64: *Replace* *“… occurs due…” by “… is largely due…”* **🡪 Ok.**

→ Lines 64-65: *Replace* *“… which feed mainly on bacteria and yeast involved in the fermentaion of each of these resources or compounds thereby made available…” by “… that feed mainly on bacteria and yeasts involved in fermentation and on fermentation by-products…”* **🡪 Ok.** **That really sounds better. Thank you.**

→ Lines 65-66: *Replace* *“… many taxa of the family…” by “… many species…”* **🡪 Ok.**

→ Line 67: *Replace* *“This is the case of…” by “This is the case for…”* **🡪 Ok.**

→ Line 68: *Replace “The Zygothrica genus group” by “The Zygothrica species group” 🡪* **This would not be correct, since the target taxon is really a genus group, as proposed by Grimaldi (1990).**

→ Line 74: *Replace* *“… reported to…” by “reported in…”* **🡪 Ok.**

→ Line 76: *Replace* *“… registered for…” by “described in…”* **🡪 Ok.**

→ Line 76: *Replace* *“… practically doubled…” by “nearly doubled…”* **🡪 Ok.**

→ Line 77: *Replace* *“… thanks to results obtained through…” by “…due to…”* **🡪 Ok.**

→ Line 77: *Replace* *“… effort…” by “… efforts…”* **🡪 Ok.**

→ Line 78: *Replace* *“In fact, this last approach also suggested…” by “The latter approach suggested…”* **🡪 Ok.**

→ Line 86: *“Nevertheless,…”* **was changed to “However,...” as requested.**

→ Line 87: *Replace* *“… paralogous…” by “… paralogues…”* **🡪 Ok.**

→ Line 88: *Replace* *“… or even due to “hitchhik…” by “… hitchhiking…”* **🡪 Ok.**

→ Line 89: *Replace* *“In fact, whereas the first phenomenon” by “The presence of numts…”* **🡪 Ok.**

→ Line 92: *Delete “Furthermore, …”* **🡪 Ok.**

→ Lines 94-95: *“… and as several species of insects can occur in a single fungus [29], mushrooms can be considered a hotspot for horizontal transfer of this endoparasite”* **was rewritten to “...therefore, mushrooms may be hotspots for horizontal transfer of this endoparasite, where multiple drosophilid species co-exist [32]”, as requested.**

→ Lines 94-97: *“In several cases, only the addition of data from nuclear gene or morphological markers could allow to assess the interference of this phenomena in the evolutionary history of the characterized mitochondrial genes”* **was rewritten to “Including nuclear gene sequences or morphological markers, along with mitochondrial genes, may allow insight into these evolutionary relationships (lines 94-97)”, as requested.**

→ Line 98: *“In this context, the aim of the present study is to confirm the subdivision of the M. projectans complex into at least three species…”* **was changed to** “**The aim of the present study was to confirm the subdivision of the *M*. *projectans* complex”, as suggested.**

→ Line 99: *“...related to their”* **was changed to “related to its”, as suggested.**

→ Line 100: *Replace* *“In this sense, we increased the sampling…” by “We increased sampling”* **🡪 Ok.**

→ Line 103: *Replace* *“These strategies…” by “These analyses…”* **🡪 Ok.**

→ Lines 105-106: *“Even so, the straightforward morphological and ecological similarity of the three species is astonishing, especially in the face of divergence dated to the Miocene.”* **was changed to “The morphological and ecological similarity of the three species was notable, especially in the face of divergence dating back to the Miocene”, as suggested.**

Materials and methods

→ Line 110: *Replace* *“… performed…” by “… made…”* **🡪 Ok.**

→ Line 111: *Replace* *“… Km^2^…” by “… km^2^…”* **🡪 Ok.**

→ Line 112: *Delete* *“… of South America area”* **🡪 Ok.**

→ Line 113: *Replace* *“… covers around…” by “… includes ca.…”* **🡪 Ok.**

→ Line 113: *Replace* *“… along Brazil’s coastal area…” by “… along coastal Brazil…”* **🡪 Ok.**

→ Lines 114-115: *Replace* *“… Rio Grande do Sul state…” by “… state of Rio Grande do Sul…”* **🡪 Ok.**

→ Line 116: *Replace* *“…tree formations…” by “… forests…”* **🡪 Ok.**

→ Line 118: *Replace* *“…adult specimens…” by “… adults…”* **🡪 Ok.**

→ Line 118: *Replace* *“…with the use of an entomological aspirator…” by “… with an aspirator…”* **🡪 Ok.**

→ Line 119: *Replace* *“…the resources were carried to the laboratory…” by “… fungi were returned to the laboratory…”* **🡪 Ok.**

→ Line 119: *Replace* *“…until adults emergence…” by “… until adults emerged…”* **🡪 Ok.**

→ Line 121: *Replace* *“Just males were employed in further analyses…” by “Only males were used…”* **🡪 Ok.**

→ Line 121-122: *“…because morphological differences in Drosophilidae are frequently described or even found through inspection of the internal genital structures of males…”* **was replaced by “because morphological differences at the aedeagus level provide more accurate species identification”, which is a little different from the Editor suggestion but still describes accurately the information.**

→ Line 128: *Delete* *“The…” at the beginning of the sentence* **🡪 Ok.**

→ Line 128: *Replace* *“… each male specimen…” by “… each adult male…”* **🡪 Ok.**

→ Line 130: *Replace* *“COI gene was…” by “COI genes were…”* **🡪 Ok.**

→ Line 142: *Add “the” before “Staden package”* **🡪 Ok.**

→ Line 143: *Replace* *“… editions were performed for…” by “… editing…”* **🡪 Ok.**

→ Line 148: *Delete* *“Additionally, for these datasets, …”* **🡪 Ok.**

→ Lines 154-155: *Replace* *“… to suggest M. projectans as…” by “… suggesting M. projectans was…”* **🡪 Ok.**

→ Line 156: *Replace* *“… in the present work…” by “… here…”* **🡪 Ok.**

→ Line 156: *Replace* *“… In the morphological approach, evaluations were performed in two ways: first, by external morphology, in which males were photographed with a stereoscopic microscope…” by “First, males were photographed with a stereoscopic microscope …” by “… here…”* **🡪 Ok.**

→ Line 157: *Replace* *“… right wings were…” by “… each right wing was…”* **🡪 Ok.**

→ Line 158: *Replace* *“… ; second, by…” by “… Second, using…”* **🡪 Ok.**

→ Lines 158-159: *Replace* *“… to search for differences in the male aedeagus structures based on Wheeler & Takada [22] descriptions…” by “… we assessed differences in male aedeagus morphology based on Wheeler & Takada…”* **🡪 Ok.**

→ Lines 159-60: *Replace* *“For this task, male terminalia was…” by “… male termnalia were…”* **🡪 Ok.**

→ Line 160: *Replace* *“… potassium hydroxide (KOH) 10% and ethanol 70%…” by “… 10% potassium hydroxide (KOH) and 70% ethanol…”* **🡪 Ok.**

→ Line 161: *Replace* *“… further disjointed…” by “… dissected…”* **🡪 Ok.**

→ Line 161: *Transfer “Leica” to the beginning of the name of the microscope* **🡪 Ok.**

→ Line 162: *Delete “To this task, …”* **🡪 Ok.**

→ Line 162: *Add “… a …” before 20x* **🡪 Ok.**

→ Line 162: *Replace* *“… under excitation wavelengths of…” by “… with a…”* **🡪 Ok.**

→ Line 163: *Replace* *“… at…” by “… to…”* **🡪 Ok.**

→ Line 163: *Delete “The evaluated morphological markers are important to Drosophilidae species delimitation and recognition and usually provide information to differentiate species [20, 22, 48-52].” at the end of the paragraph* **🡪 Ok.**

→ Line 164: *Replace* *“Under the molecular approach, all individuals were first…” by “… all individuals were…”* **🡪 Ok.**

→ Line 167: *Replace* *“… the reciprocal monophyly…” by “… reciprocal monophyly…”* **🡪 Ok.**

→ Lines 173-174: *Replace* *the* *“[…]” by “, …,” for the text explaining precedence of the sequence MycoNova* **🡪 Ok.**

→ Line 180: *Add “the” before “Stacey package”* **🡪 Ok.**

→ Line 186: *Add “the” before “Splits package”* **🡪 Ok.**

→ Line 192: *Replace* *“200.000.000” and “10.000” by “200,000,000” and “10,000” respectively* **🡪 Ok.**

→ Line 194: *Replace* *“… specimens assignments…” by “… species assignments…”* **🡪 Ok.**

→ Line 202: *Replace* *“… intra…” by “… intra-…”* **🡪 Ok.**

→ Line 208: *Delete “For the morphometric analysis, …” at the beginning of the paragraph* **🡪 Ok.**

→ Line 208: *Replace* *“… male specimens…” by “…males…”* **🡪 Ok.**

→ Line 208: *Replace* *“… were photographed in…” by “…were photographed with…”* **🡪 Ok.**

→ Line 210: *Delete “To obtain the coordinates, …” at the beginning of the sentence* **🡪 Ok.**

→ Lines 210-211: *Replace* *“… chosen with basis on wings venation…” by “…chosen based on wing venation…”* **🡪 Ok.**

→ Line 212: *Add* *“a” before “with matrix”* **🡪 Ok.**

→ Lines 213-214: *Replace* *“An analysis of variance…” by “Analysis of variance…”* **🡪 Ok.**

→ Line 225: *Replace* *“An analysis of molecular variance…” by “Analysis of molecular variance…”* **🡪 Ok.**

→ Lines 213-214: *Replace* *“…performed in the same software…” by “…performed with the same software…”* **🡪 Ok.**

→ Line 236: *Correct* *“coalescent”* **🡪 Ok.**

→ Line 243: *Replace* *“… within Drosophilidae phylogeny…” by “…within the Drosophilidae…”* **🡪 Ok.**

→ Lines 247-250: *Replace* *the* *“[…]” by “, …,” for the text explaining the priors and include these within the main text with expressions like “using a prior with a mean of …” or “with a prior with a mean of…”* **🡪 Ok.**

→ Line 252: *Replace* *“… ploidy level…” by “… ploidy levels…”* **🡪 Ok.**

→ Line 252: *Replace* *“… presented by…” by “… for…”* **🡪 “… presented by” was replaced by “of”.**

→ Line 254: *Replace* *“… burning…” by “… burn-in…”* **🡪 Ok.**

→ Line 254: *Delete “After, …” at the beginning of the sentence* **🡪 Ok.**

→ Line 254: *Replace* *“… the run was…” by “… the run was then…”* **🡪 Ok.**

→ Line 264: *Replace* *“10.000” by “10,000”* **🡪 Ok.**

→ Line 264: *Replace* *“… burn in the first…” by “burning the first”* **🡪 Ok.**

→ Line 276: *Replace* *“As the cryptic diversity contained within the M. projectans complex was previously neglected in the literature” by “As cryptic diversity in the M. projectans species complex was previously neglected…”* **🡪 Ok.**

→ Line 277: *Replace* *“… recorded by…” by “… recorded in…”* **🡪 Ok.**

→ Line 277: *Delete “… in this analysis” at the end of the sentence* **🡪 Ok.**

→ Line 277: *Replace* *“… presence point…” by “… sampling point…”* **🡪 Ok.**

→ Line 280: *Add* *“the” before “dismo package”* **🡪 Ok.**

→ Line 288: *Replace* *“… Schoener’s (D) and Hellinger’s (I) …” by “… Schoener’s D and Hellinger’s I …”* **🡪 Ok.**

→ Line 289: *Replace* *“… are…” by “… were…”* **🡪 Ok.**

Results

→ Line 295: *Delete* *“In the present study…” in the beginning of the section* **🡪 Ok.**

→ Line 296: *Replace* *“… number…” by “… numbers…”* **🡪 Ok.**

→ Line 299: *Replace* *“No sequence presented…” by “No sequences contained…”* **🡪 Ok.**

→ Lines 301-302: *Replace* *“… presented low K2P distance” by “… showed low K2P distances”* **🡪 Ok.**

→ Notes of Table 1: *Replace* *“heterozigosity” by “… heterozygosity”* **🡪 Ok.**

→ Line 305: *Replace* *“How many species are there under the general M. projectans morphotype” by “How many M. projectans species are there”* **🡪 Ok.**

→ Line 307: *Replace* *“… the 172 COI sequences” by “… of the 172 COI sequences”* **🡪 Ok.**

→ Line 307: *Replace* *“:” by “,”* **🡪 Ok.**

→ Line 309: *Replace* *“[ ]” by “,”* **🡪 Ok.**

→ Line 309: *Replace* *“The total set of specimens…” by “All individuals”* **🡪 Ok.**

→ Line 311: *Replace* *“;” by “,”* **🡪 Ok.**

→ Line 312: *Replace* *“Moreover, to the exception of…” by “With the exception of…”* **🡪 We preferred to adopt a more concise form “Except for”**

→ Line 313: *Replace* *“… the three species revealed…” by “… the three species were…”* **🡪 Ok.**

→ Line 324: *Replace* *“… for the COI marker…” by “… for COI…”* **🡪 We transferred the name of the marker, which is now mentioned regarding “maximum COI intraspecific distances”.**

→ Line 324: *Replace* *“… presented…” by “… showed…”* **🡪 Ok.**

→ Line 325: *Delete* *“On the other hand…”* **🡪 Ok.**

→ Line 329: *Replace* *“… presented…” by “… showed…”* **🡪 Ok.**

→ Line 332: *Replace* *“… is…” by “… was…”* **🡪 Ok.**

→ Line 334: *Replace* *“Although at a first sight specimens” by “… Individuals…”* **🡪 Ok.**

→ Line 334: *Replace* *“… are…” by “… were…”* **🡪 Ok.**

→ Line 334: *Add* *“… as…” before “… we could identify…”* **🡪 We preferred to add “but” instead of “as”, since the sentence presents an opposing argument.**

→ Line 335: *Replace* *“… two patterns shared…” by “… Two patterns were shared…”* **🡪 Ok.**

→ Line 337: *Replace* *“… are…” by “… were…”* **🡪 Ok.**

→ Lines 337-339: *Replace the “( )” by “,” to delimit the sentences “hereafter called abdominal pattern x”* ***🡪* Ok.**

→ Line 341: *Replace* *“… with exceptions to this pattern found in two individuals attributed to this species…” by “There were two exceptions to this pattern found in this species…”* **🡪 Ok.**

→ Line 344: *Replace* *“… confirms a close resemblance…” by “… confirmed the close resemblance…”* **🡪 Ok.**

→ Line 346: *Delete* *“The” before “wing” at the beginning of the sentence* **🡪 Ok.**

→ Line 346: *Replace* *“… tested…” by “… used…”* **🡪 Ok.**

→ Line 346: *Replace* *“… 57 specimens…” by “… 57 individuals…”* **🡪 Ok.**

→ Line 350: *Delete* *“as” before “a wide overlap”* **🡪 Ok.**

→ Lines 351-352: *Replace* *“… wings did not provide adequate resolution…” by “… wing morphology did not provide enough resolution…”* **🡪 Ok.**

→ Line 353: *Replace* *“At the male genital levels…” by “At the male genitalia levels…”* **🡪 Ok.**

→ Line 357: *Replace* *“… shared…” by “… was shared…”* **🡪 Ok.**

→ Line 360: *Replace* *“… with aedeagus longer…” by “… with the aedeagus longer…”* **🡪 We preferred to replace the previous expression by “with a longer aedeagus”, because it fits better with the next reasoning.**

→ Line 367: *“… even the mitochondrial markers…”* **was replaced by “… even both mitochondrial markers…”, because the suggestion would change the meaning of the sentence.**

→ Line 368: Replace *“… as sister species…” by “… as a sister species”* **🡪 We think this suggestion would not be adequate within the arrangement of the ideas.**

→ Line 374: Replace *“… whereas the next cladogenesis occurred shortly after, around 15 Mya.” by “… and the next split occurred ca. 15 Mya”* **🡪 Ok.**

→ Lines 376-378: *The subtitle “What can we say about the differential patterns of genetic diversity and abiotic niche that distinguish the three species and what this suggests about their speciation”* **was replaced by “Patterns of distribution of genetic diversity and abiotic niches help to disentangle evolutionary history and suggest modes of speciation”, as suggested.**

→ Line 380: *Delete “as” before “for mitochondrial and nuclear markers, respectively”* ***🡪* Ok.**

→ Lines 380-381: *Replace the “( )” by “,” to delimit the sentence “for mitochondrial and nuclear markers, respectively”* ***🡪* Ok.**

→ Line 382: *Replace “presented” by “revealed”* ***🡪* Ok.**

→ Line 382: *Replace “attained” by “showed”* ***🡪* Ok.**

→ Line 388: *Replace “is” by “was”* ***🡪* Ok.**

→ Line 390: *Replace “is commonly very similar” by “was very similar”* ***🡪* Ok.**

→ Lines 392-393: *Replace the “… presented a star-like pattern…” by “showing star-like patterns”* ***🡪* Ok.**

→ Line 394: *Replace “are” by “were”* ***🡪* Ok.**

→ Line 398: *Replace “haplotype” by “haplotypes”* ***🡪* Ok.**

→ Line 399: *Replace “Although the differentiation…” by “Although differentiation…”* ***🡪* Ok.**

→ Line 400: *Replace “… seems to be maintained…” by “… seems to have been maintained…”* ***🡪* Ok.**

→ Line 406: *Replace “is” by “was”* ***🡪* Ok.**

→ Line 409: *Replace “… sampled in the border…” by “… sampling along the border…”* ***🡪* Ok.**

→ Line 409: *Add “The” before “Atlantic Forest”* ***🡪* Ok.**

→ Line 410: *Replace “… present…” by “… contained…”* ***🡪* Ok.**

→ Line 411: *Replace “… radiates…” by “… radiated…”* ***🡪* Ok.**

→ Lines 417-418: *Delete the information presented in parenthesis for the subdivision of M. projectans affinis 1 and affinis 3* ***🡪* Ok.**

→ Lines 419-420: *Replace the “( )” by “,” to delimit the sentence “with two groups composed of individuals from the Atlantic Forest and another group exclusively composed of specimens from the Pampa biome”* ***🡪* Ok.**

→ Line 419: *Replace “… specimens…” by “… individuals…”* ***🡪* Ok.**

→ Line 419: *Add “The” before “Atlantic Forest”* ***🡪* Ok.**

→ Line 421: *Replace “… presented…” by “… revealed…”* ***🡪* Ok.**

→ Line 424: *Add “The results of…” before “the Extended Bayesian Skyline”* ***🡪* Ok.**

→ Line 424: *Replace “… seems to agree…” by “… agreed…”* ***🡪* Ok.**

→ Line 425: *Add “the” before “COI networks”* ***🡪* Ok.**

→ Line 429: *Replace “… ranges…” by “… ranged…”* ***🡪* Ok.**

→ Line 434: *Replace “… showed to be…” by “… are…”* ***🡪* Ok.**

→ Line 434: *Replace “… to an area…” by “… in an area…”* ***🡪* Ok.**

→ Line 435: *Replace “… Southern…” by “… southern…”* ***🡪* Ok.**

→ Line 435: *Delete “… their distribution can go much further, and specimens identified as…”* ***🡪* Ok.**

→ Line 436: *Replace “… have…” by “… have…”* ***🡪* Ok.**

→ Line 437: *Replace “… sampled area…” by “… areas sampled…”* ***🡪* Ok.**

→ Line 437: *Replace “… exploring fungi…” by “… associated with fungal…”* ***🡪* Ok.**

→ Line 438: *Replace “… the three species…” by “… all three species…”* ***🡪* Ok.**

→ Line 442: *Add “… in the sampled genera…” before Ganoderma* ***🡪* We inserted “in species of”.**

→ Lines 444-445: *Replace “. According to these results, although…” by “, suggesting…”* ***🡪* We preferred to separate the ideas in two sentences. Thus, we changed the subject of the second sentence, and the verb was conjugated to “suggested”.**

→ Lines 445-446: *Replace “… population overlaps in Southern Brazil are plausible not only for the present but also for different moments of the past…” by “… with present overlapping ranges in Southern Brazil and possibly at different times in past…”* ***🡪* We replaced previous sentence by “with present and past overlapping ranges”.**

→ Line 447: *Add “… the…” before Pleistocene* ***🡪* Ok.**

→ Line 452: *Delete “Nevertheless…” in the beginning of the sentence* ***🡪* Ok.**

→ Line 455: *Replace “Anyway…” by “Thus…”* ***🡪* We replaced “Anyway…” by “However…”, because these are contrasting results.**

Discussion

→ Line 459: *Delete “DNA Barcoding proved here to be a great tool for the identification and discovery of species and for understanding diversity in the Zygothrica genus group, as previously shown by Machado et al. [25]. Moreover…” at the beginning of the paragraph* ***🡪* Ok.**

→ Line 459: *Replace “… presents…” by “… revealed…”* ***🡪* Ok.**

→ Line 459: *Replace “… suggested…” by “… hypothesized…”* ***🡪* Ok.**

→ Line 460: *Replace “… a high incidence…” by “… a number…”* ***🡪* We used “several” instead of “a number of” to favor a concise language.**

→ Line 460: *Delete “Nevertheless…” at the beginning of the sentence* ***🡪* Ok.**

→ Line 463: *Replace “…hitchhike with the selective pressure acting on…” by “..hitchhiking due to”* ***🡪* Ok.**

→ Line 464: *Replace “… both artifacts…” by “both factors”* ***🡪* Ok.**

→ Line 464: *Replace “… proposition of the three species of the M. projectans complex…” by “… proposed inclusion of three species in the M. projectans complex”* ***🡪* Ok.**

→ Line 465: *Replace “… discarded…” by “… discounted…”* ***🡪* Ok.**

→ Lines 465-466: *Replace “… by the inclusion of evidence provided by this study…” by “… by the evidence provided in the current study…”* ***🡪* Ok.**

→ Line 467: *Delete “At first, the…” at the beginning of the paragraph* ***🡪* Ok.**

→ Line 467: *Replace “… diminishes…” by “… were diminished…”* ***🡪* Ok.**

→ Line 472: *Replace “… in our dataset…” by “… here…”* ***🡪* Ok.**

→ Line 473: *Replace “… it was possible to show that reciprocal monophyly and…” by “… we showed that reciprocal monophyly was consistent with…”* ***🡪* Ok.**

→ Line 477: *Replace “… occurs…” by “… occurred…”* ***🡪* Ok.**

→ Line 478: *Replace “In this study, besides…” by “In addition to…”* ***🡪* Ok.**

→ Line 478: *Delete “… by both mitochondrial markers…” and quote only “COI and COII”* ***🡪* Ok.**

→ Line 480: *Insert “the” before “ABGD”* ***🡪* Ok.**

→ Line 483: *Replace “… allowed rejecting…” by “… allowed us to reject…”* ***🡪* Ok.**

→ Line 493: *Delete “… at a first sight…” after “Although”* ***🡪* Ok.**

→ Lines 494-495: *“… shortages related to the amplification success presented by some of the markers employed here would not allow a confident description of prevalence patters with such a metodology”* **was replaced by “shortages related to the amplification success of some of our markers showed that such estimates of infection rates would not be precise”.**

→ Line 496: *Replace “… if…” by “… whether…”* ***🡪* Ok.**

→ Line 496: *Replace “… could or not be…” by “… was…”* ***🡪* Ok.**

→ Line 496: *Replace “… speciation…” by “… diversification…”* ***🡪* Ok.**

→ *Delete fourth and fifth paragraphs of the discussion* ***🡪* Ok. Please see that ideas previously presented on the sixth paragraph now appear on the third paragraph of the discussion.**

→ Line 504: *Replace “… sampling points located in…” by “… samples from…”* ***🡪* Ok.**

→ Line 505: *Delete “However, as sampling in several areas was not extensive, both sympatry and sintopy can be even more ubiquitous than shown”* ***🡪* Ok.**

→ Line 509: *Delete “This may suggest that species recognition and preferential mating may be acting at levels other than visual, as previously observed in Drosophila silvestris”* ***🡪* Ok.**

→ Line 510: *Replace “… biotic and abiotic niche…” by “… biotic and abiotic niches…”* ***🡪* Ok.**

→ Line 510: *Replace “… fungus…” by “… fungi…”* ***🡪* Ok.**

→ Line 511: *Delete “Thus,…” before despite* ***🡪* Ok.**

→ Line 514: *Delete “…, in agreement with Robe et al.” after referring to the hypothesis of niche conservatism for the M. projectans complex****🡪* Ok.**

→ Line 516: *Replace “… in which way the…” by “… how these…”* ***🡪* Ok.**

→ Line 517: *Delete “Interestingly,…” at the beginning of the paragraph* ***🡪* Ok.**

→ Line 516: *Replace “… the pattern…” by “Patterns…”* ***🡪* Ok.**

→ Line 517: *Replace “… divergence…” by “… divergences…”* ***🡪* Ok.**

→ Line 517: *Delete “This divergence timing was established using a fossil calibration [79,80], and two contrasting molecular clock estimates provided by Tamura et al. [81] and Suvorov et al. [82] for the divergence between Sophophora and the remaining Drosophila. The first of these studies employed 176 nuclear genes to estimate a genomic mutation clock rate, using the time of formation of Kauai Island in Hawaii as a prior to the divergence of D. picticornis and other species of the planitibia group of Hawaiian drosophilids [97]. Suvorov et al. [82] employed 155 genome assemblies to generate a matrix of 2,791 genes, which was used to reconstruct a fossil calibrated phylogeny, based on at least five fossil datings”* ***🡪* Ok.**

→ Line 517: *Replace “… underestimation…” by “… underestimations…”* ***🡪* The entire sentence was withdrawn in this reviewed version.**

→ Line 522: *Replace “According to our estimates,…” by “Our estimates indicated…”* ***🡪* Ok.**

→ Line 526: *Delete “Such a result may be explained by the presence of nonvisual mating signals and/or by the action stabilizing selection promoting morphological stasis [103].” Before Furthermore* ***🡪* Ok.**

→ Line 529: *Replace “… alo…” by “… -alo…”* ***🡪* Ok.**

→ Line 530: *Replace “… is…” by “… was…”* ***🡪* Ok.**

→ Line 531: *Replace “… for all the evaluated species” by “… for all species…”* ***🡪* Ok.**

→ Line 537: *Replace “In agreement with this…” by “Further…”* ***🡪* Ok.**

→ Line 537: *Replace “… the…” by “… these…”* ***🡪* Ok.**

→ Line 538: *Replace “… as evidenced…” by “… as shown…”* ***🡪* Ok.**

→ Line 538: *Replace “The high influence…” by “The influence…”* ***🡪* Ok.**

→ Line 539: *Replace “… supports…” by “… supported…”* ***🡪* Ok.**

→ Line 540: *Replace “… of these three species…” by “… of these species…”* ***🡪* Ok.**

→ Line 541: *Transfer “… in the Southern Hemisphere…” to the end of this sentence* ***🡪* Ok.**

→ Line 549: *Replace “… presenting evidence…” by “… suggesting…”* ***🡪* Ok.**

→ Line 550: *Replace “… to the…” by “… in…”* ***🡪* Ok.**

→ Line 550: *Replace “… further samplings are…” by “… further sampling is…”* ***🡪* Ok.**

→ Line 553: *Delete “Interestingly, …” at the beginning of the sentence* ***🡪* Ok.**

→ Line 553: *Delete “… in the sampled region …” after diversification* ***🡪* Ok.**

→ Line 553: *Delete “… (specially M. projectans affines 3) …” after complex* ***🡪* Ok.**

→ Line 553: *Replace “… show…” by “… showed…”* ***🡪* Ok.**

→ Line 554: *Replace “Southernmost” and “biomes” by “southernmost” and “biomes”* ***🡪* Ok.**

→ Line 554: *Add “the” before “Pampa”* ***🡪* Ok.**

→ Line 555: *Delete “This suggests the existence of intrinsic adaptations related to each of these habitats, which, to our knowledge, were not previously found for other Drosophilidae species. …” before “The levels…”****🡪* Ok.**

→ Line 556: *Replace “… for the three species of the…” by “… for the…”* ***🡪* Ok.**

→ Line 556: *Replace “… are…” by “… were…”* ***🡪* Ok.**

→ Line 559: *Replace “… presented…” by “… showed…”* ***🡪* Ok.**

→ Line 559: *Delete “Although, these high diversity indices could suggest co-amplification of numts, tests performed to identify this artifact did not detect such an effect…” before “This high diversity…”****🡪* Ok.**

→ Line 559: *Replace “… presented…” by “… showed…”* ***🡪* Ok.**

→ Line 562: *Delete “… in the haplotype networks of M. projectans affinis 2, it is possible to notice …” after “In this sense, …” and add this idea to the end of the sentence (lines 564-565)****🡪* Ok.**

→ Line 563: *Replace “… are…” by “… were…”* ***🡪* Ok.**

→ Line 563: *Replace “… closer in relation to…” by “… more similar in…”* ***🡪* Ok.**

→ Line 565: *Replace “This suggests a metapopulation dynamics…” by “This suggests a possible metapopulation dynamics…”* ***🡪* Ok.**

→ Line 565: *Delete “…, which was already” after “dynamics”* ***🡪* Ok.**

→ Line 566: *Replace “… in virtue of…” by “… due to…”* ***🡪* We replaced the stretch by “in response to”.**

Conclusion

→ Line 574: *Replace “… exclude interferences such as numts…” by “… exclude numts…”* ***🡪* Ok.**

→ Line 575: *Replace “… was dated…” by “… dated…”* ***🡪* Ok.**

→ Line 577: *Replace “… the abiotic niche was identical…” by “… abiotic niches were equivalent…”* ***🡪* Ok.**

→ Line 578: *Replace “… generally presented…” by “… showed…”* ***🡪* Ok.**

→ Line 579: *Delete “In this sense,…” in the beginning of the last sentence* ***🡪* Ok.**

→ Line 580: *Replace “… this study certainly represents…” by “… this study represents…”* ***🡪* Ok.**

→ Line 581: *Replace “… comprehension of the spatio-temporal…” by “… understanding of potential spation-temporal…”* ***🡪* Ok.**
